# Optimization of Roughness, Dimensional Conformity, and Porosity of 3D-Printed ASA with MEX: Impact of Critical Process Control Parameters

**DOI:** 10.1021/acsomega.5c07437

**Published:** 2025-11-18

**Authors:** Dimitrios Sagris, Constantine David, Markos Petousis, Nektarios K. Nasikas, Nikolaos Mountakis, Maria Spyridaki, Nectarios Vidakis

**Affiliations:** † Dept. of Mechanical Engineering, 125444 International Hellenic University, Serres Campus, Thessaloniki 62124, Greece; ‡ Dept. of Mechanical Engineering, 112178 Hellenic Mediterranean University, Heraklion 71410, Greece; § Division of Mathematics and Engineering Sciences, Department of Military Sciences, 69139 Hellenic Army Academy, 16673 Vari, Attica, Greece

## Abstract

Acrylonitrile–styrene–acrylate (ASA) is a material with high potential, making it suitable for outdoor applications, which warrants further investigation. Its presence in additive manufacturing (AM) shows potential. Herein, ASA three-dimensional (3D) printed samples are examined using a Taguchi L25 experimental design to achieve optimum quality characteristics, through the improvement of the performance across multiple response metrics, including root-mean-square roughness (*R*_q_), average roughness (*R*_a_), actual-to-nominal dimensional deviation (A2N_95_) and CT scan porosity (*P*_CT_). Six variable control parameters were examined: extrusion width, raster orientation, layer height, deposition velocity, extruder temperature, and substrate temperature. Experimental findings showed that *R*_a_ and *R*_q_ can be improved by more than 250% (*R*_a_ reduced from 17.37 to 6.79 μm, *R*_q_ reduced from 21.71 to 9.29 μm), geometrical accuracy can be enhanced by 324% (A2N_95_ reduced from 437.76 134.95 μm), and porosity can be reduced by 564% (PCT reduced from 9.08 to 1.61%) when selecting a proper set of 3D printing settings. Different regression models were evaluated: the reduced quadratic (RQRM), linear (LRM), and quadratic (QRM) regression models. LRM was inferior, while RQRM and QRM had remarkably close prediction accuracies; thus, the RQRM was proposed for this experimental scenario. The two confirmation runs yielded prediction equations with an error of less than 10% between the predicted and calculated values. The extruder temperature and extrusion width were the two parameters causing the greatest impact on the responses.

## Graphical abstract

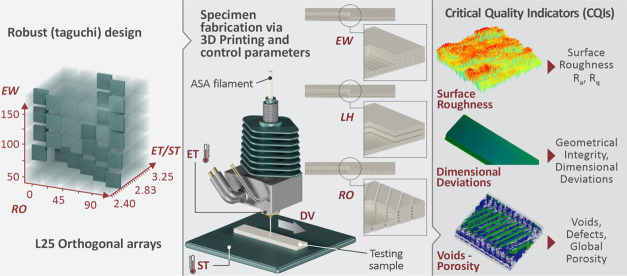

## 1 Introduction

Additive manufacturing (AM) offers a range of technologies^1^ capable of meeting the growing demand for desired products and components, especially in 3D printing.^2^ The 3D printing method is a repeated layer-deposition procedure that can produce parts of various shapes and structures, regardless of complexity.^3,4^

Simultaneously, cost effectiveness,^5,6^ waste^7,8^ and energy reduction,^9,10^ and ease of manufacturing^11–13^ are significantly enhanced.^14^ The applications of additive manufacturing extend across diverse industries, including aerospace,^15,16^ automotive,^17,18^ healthcare and medical,^19–22^ fabric and fashion,^23^ electrical^24,25^ and electronics,^26,27^ among others.^1,11,28^ Food processing has also demonstrated potential for taking advantage of 3D printing.^29,30^

AM utilizes a great variety of materials, such as ceramics^31^ (e.g., Al_2_O_3_,^32^ SiO_2_,^33^ Si_3_N_4_ ^34^), metals^35^ (e.g., silver,^36^ titanium,^37^ gold^38^), polymers,^21,39,40^ high- and ultrahigh-performance polymers,^41,42^ composites,^43–45^ and blends.^46–48^ However, polymers are considered the most utilized materials because of their ease of printing and cost effectiveness.^49^

The most commonly utilized polymeric materials in AM are acrylonitrile–butadiene–styrene (ABS),^50–53^ polypropylene (PP),^54–57^ high-density polyethylene (HDPE),^58–60^ polyamides,^61–63^ acrylonitrile–styrene (ASA),^64–66^ polyethylene terephthalate (PET),^67–69^ polylactic acid (PLA),^70–72^ polycarbonate (PC),^73–75^ polyethylene terephthalate glycol (PETG),^76–78^ polyether ether ketone (PEEK),^79–81^ etc.^82^

ASA is an amorphous thermoplastic material.^83^ It is considered to have characteristics,^84,85^ mechanical properties, and printing conditions similar to those of the ABS material.^86^ It is reported to be a sustainable thermoplastic, as it can be reused after multiple thermal reprocessing cycles (in 3D printing as well).^87^ It possesses environmental stability,^86^ as well as great ultraviolet (UV), weather, and oil-heat resistance,^88,89^ with a glass transition temperature (*T*_g_) of 118.5 °C.^90^ Its unique properties and ability to withstand harsh conditions make it a material of great research interest^91,92^ and preferable for outdoor applications.^93,94^ It is used in the automotive,^95–97^ electronics, and electrical^98^ sectors.

It should be noted that ASA material is regularly combined with ABS to create 3D-printed composite parts.^99^ It has been proven that the mechanical properties of ASA are improved when it is combined with ABS.^90^ ASA mechanical properties in 3D-printed parts have been reported and optimized previously.^100^ An L18 orthogonal array Taguchi experimental design was used to optimize the mechanical properties of the ASA samples.^101^ The greatest influence was revealed by the layer height and infill density, while the maximum tensile, flexural, and impact strengths were measured to be 51.86 MPa, 82.56 MPa, and 0.180 J/mm^2^, respectively.

The influence of 3D printing settings on the tensile properties of 3D-printed ASA using the material extrusion (MEX) method has also been investigated.^86^ Central composite design and response surface methodology were used in this study, and the results indicated a significant influence of the layer thickness parameter. The influence of printing parameters on 3D-printed ASA specimens has also been reported in other research.^102^ It has been reported that ASA 3D-printed parts can also be efficiently exploited in hybrid AM methods.^103^ Previous optimization studies using Taguchi or regression modeling for AM in ASA, and related to quality aspects, employ laser cutting to improve the quality of the parts, along with machine learning approaches.^104^ Related studies have been conducted on ABS thermoplastic, which exhibits similar characteristics to ASA.^105^

Considering the ASA market value, it is poised to grow, as indicayed by available related reports.^106–109^ Previous research reported on the ASA 2024–2034 forecast period, stating an 8.9% compound annual growth rate (CAGR), as the market size was expected to expand from USD 1 billion to USD 1.71 billion.^110^ GNITIVE Market Research reported a 6.2% CAGR between 2023 and 2030, from USD 858.2 million in the year 2023.^111^

The above bibliography reveals that research in ASA in AM has focused mainly so far on its mechanical properties, and this area can also be considered rather limited. One critical aspect of the 3D-printed parts is their quality, which is affected by high surface roughness, geometrical deviation from the nominal values, and porosity of the 3D printing structure.^112–117^ These quality aspects affect not only the functionality of the 3D-printed parts but also their performance.^118–121^ For example, high porosity can lead to reduced tensile strength, stiffness, and impact strength.^118^ Surface roughness can initiate stress concentration, leading to premature crack initiation, especially in tensile or fatigue loadings. Therefore, improving these quality aspects is crucial for the performance of 3D-printed parts. Although such investigations have been presented for different thermoplastics in AM, as mentioned,^112–117^ even for the ABS thermoplastic (which is similar to ASA), or PLA, which is the most popular thermoplastic in AM, no research was found in the bibliography search for the ASA polymer.

The novelty of the research lies in the application of a Taguchi design of experiments, in conjunction with regression analysis, for the simultaneous optimization of porosity, surface roughness, and dimensional accuracy of 3D-printed ASA parts built using MEX methods. This represents a systematic, multiobjective approach to elucidating complex process–structure–property relationships. This methodology encompasses a comprehensive strategy aimed to capture the interacting and contrasting relationships among six correlated printing parameters, considering both synergistic and antagonistic influences. The utilization of the Taguchi method offers a statistically robust framework for identifying the most significant control factors for each response and calculating the signal-to-noise ratio to differentiate between optimal settings for each factor with a target value and those that minimize variations. From this empirical data, regression models are developed to provide a predictive mathematical framework, enabling the quantification of nonlinear covariations and the estimation of output characteristics across the design space with a reliable level of confidence. Consequently, the primary innovation of this investigation lies in the development of a validated, computationally efficient optimization protocol that aims to replace the iterative trial-and-error approach with a deterministic process. This process facilitates the attainment of the best possible compromise between competing quality characteristics, ultimately enhancing the manufacturability and reliability of ASA parts for functional end-use applications in demanding environments.

Herein, the aim is to optimize the root-mean-square roughness (*R*_q_), average roughness (*R*_a_), actual-to-nominal dimensional deviation (A2N_95_), and CT scan porosity (*P*_CT_) of 3D-printed ASA samples using the MEX AM technique by optimizing the 3D printing parameters, such as extrusion width (EW), raster orientation (RO), layer height (LH), deposition velocity (DV), extruder temperature (ET), and substrate temperature (ST). For this purpose, the Taguchi L25 design of experiment (DOE) was employed. The linear regression model (LRM), quadratic regression model (QRM), and reduced quadratic regression model (RQRM) were the three regression models utilized for the extraction of the respective prediction models. Such an approach is common in experimental data analysis and optimization and has proven effective in AM previously.^122–125^ Taguchi’s design of experiments method enables the systematic identification of the most critical parameters affecting the response metrics while significantly reducing reducing the number of experimental runs. Regression modeling facilitates the development of predictive equations that express the complex relationships between process control parameters and the quality metrics of 3D-printed parts. This approach introduces a robust framework for optimizing MEX printing of ASA components, addressing critical quality aspects that impact mechanical performance, as mentioned. This research advances the field by offering a quantitative and scalable methodology tailored for ASA in MEX 3D printing and thus promotes the quality of fabricated components and their functionality in AM applications.

## 2 Materials and Methods

### 2.1 Materials

The company 3DXtech (Grand Rapids, Michigan, USA) supplied the ASA raw material (grade 3DXmax). The provided datasheet specifies the material features, including a density of 1.07 g/cm^3^ and a glass transition temperature of 105 °C.

### 2.2 Experimental Procedure

Figure 1 shows the experimental modeling strategy in the flowchart form, described in the left section. In the experimental strategy flowchart, the variable control parameters, fixed control parameters, and output metrics are specified. The main experimental procedures of this study are illustrated in the right section. The experimental procedure steps depicted in the right section include material drying in the oven (Figure 1a), extrusion of the filament and drying (Figure 1b,c), 3D printing and optical microscopy evaluation of the coupons (Figure 1d,e), optical profilometry, and micro-CT scanning (Figure 1f,g).

**Figure 1:**
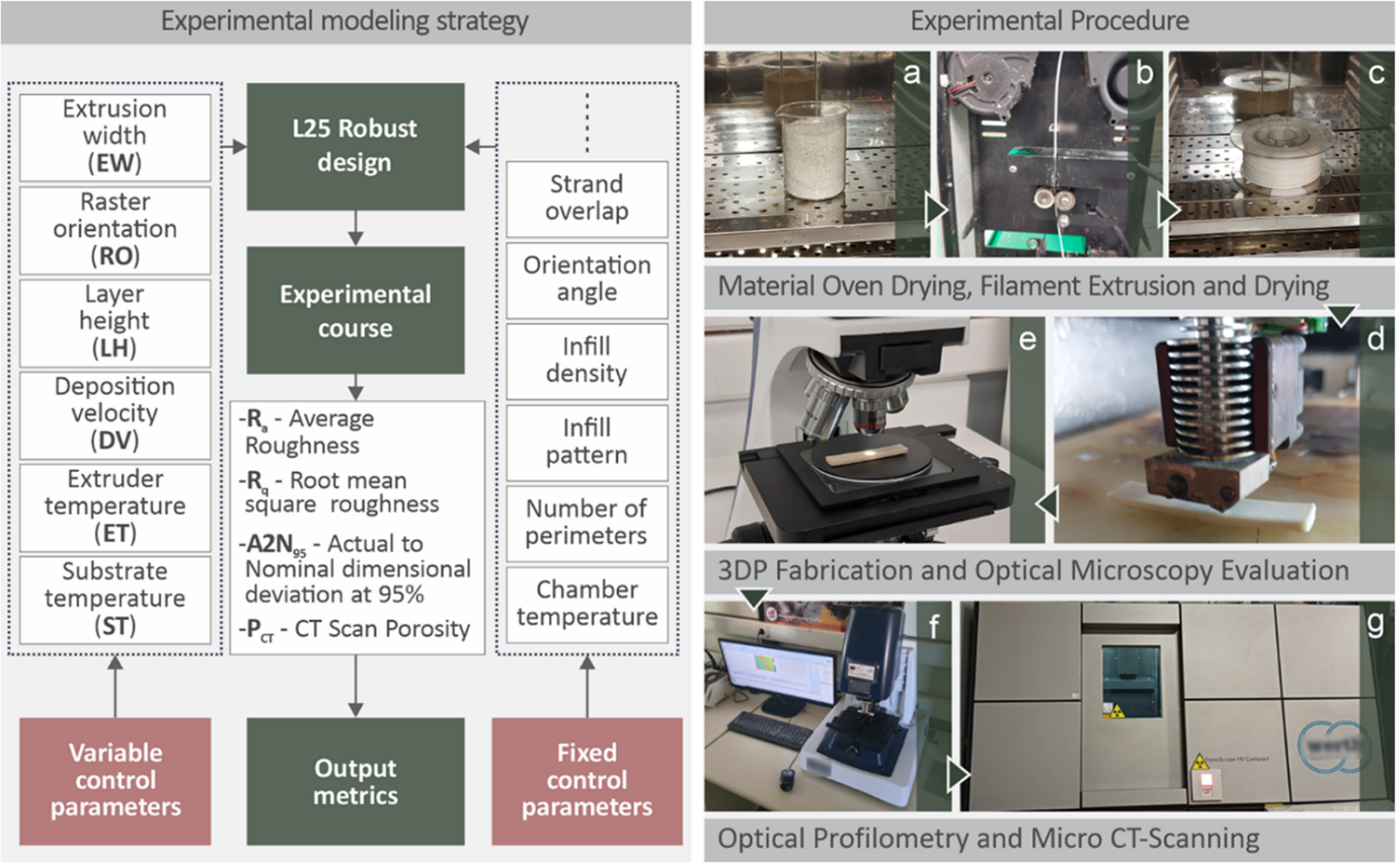
Experimental modeling strategy (left section) and images of the main experimental procedures (right section): (a) drying of the raw material, (b) extrusion of the filament, (c) drying of the filament, (d) coupon fabrication (3D printing), (e) and (f) optical microscopy images, and (g) microcomputed tomography scans.

### 2.3 ASA Thermal, Viscosity, and Raman Assessment

The ASA thermal-related information is valuable for the determination of possible material degradation caused by the applied temperatures. Thermogravimetric analysis (TGA) and differential scanning calorimetry (DSC) were conducted by using a PerkinElmer Diamond (Waltham, Massachusetts) and a TA Instruments Discovery-Series DSC 25 (Delaware) apparatus. Figure 2 shows the TGA curves, 3D printing temperatures, initial decomposition temperature (IDT), final residue (FR) (Figure 2a), DSC endothermic/heating curves, glass transition temperature (*T*_g_), 3D printing temperature range (Figure 2b), and exothermic cooling curves (Figure 2c). Moreover, viscosity results were obtained (Figure 2d) along with Raman examination results (Figure 2e). The information provided in Figure 2a–e was derived from the ASA pure sample data of previously conducted research.^126^

**Figure 2:**
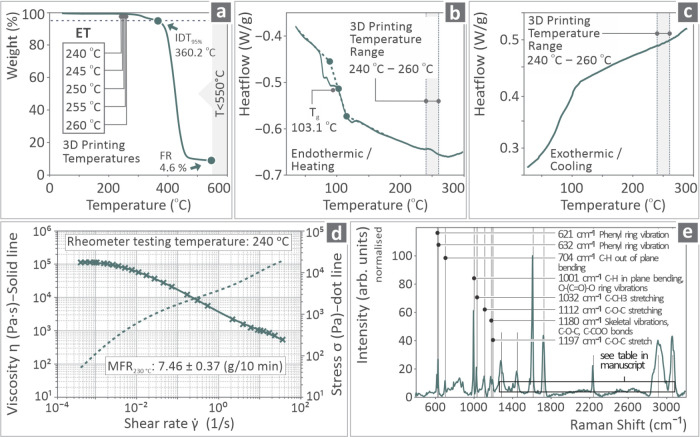
(a) TGA curves, (b) DSC endothermic/heating and (c) exothermic heating curves, (d) viscosity graph, and (e) Raman spectra.

### 2.4 ASA 3D Coupon Printing

For manufacturing the specimens, MEX 3D printing was utilized (Funmat HT, Intamsys, Shanghai, China). The selected control parameters are illustrated in Figure 3a. Their levels are shown in Figure 3b, while Figure 3c shows the dimensions of the test specimen. The three raster orientation values examined are shown in the sample design. The specimen’s geometry was derived from the ASTM D790 international standard (bending test specimen). Samples were prepared according to ASTM D790 for reference puropses only, to standardize sample shape. Testing their mechanical performance in such tests was not within the scope of this research.

**Figure 3:**
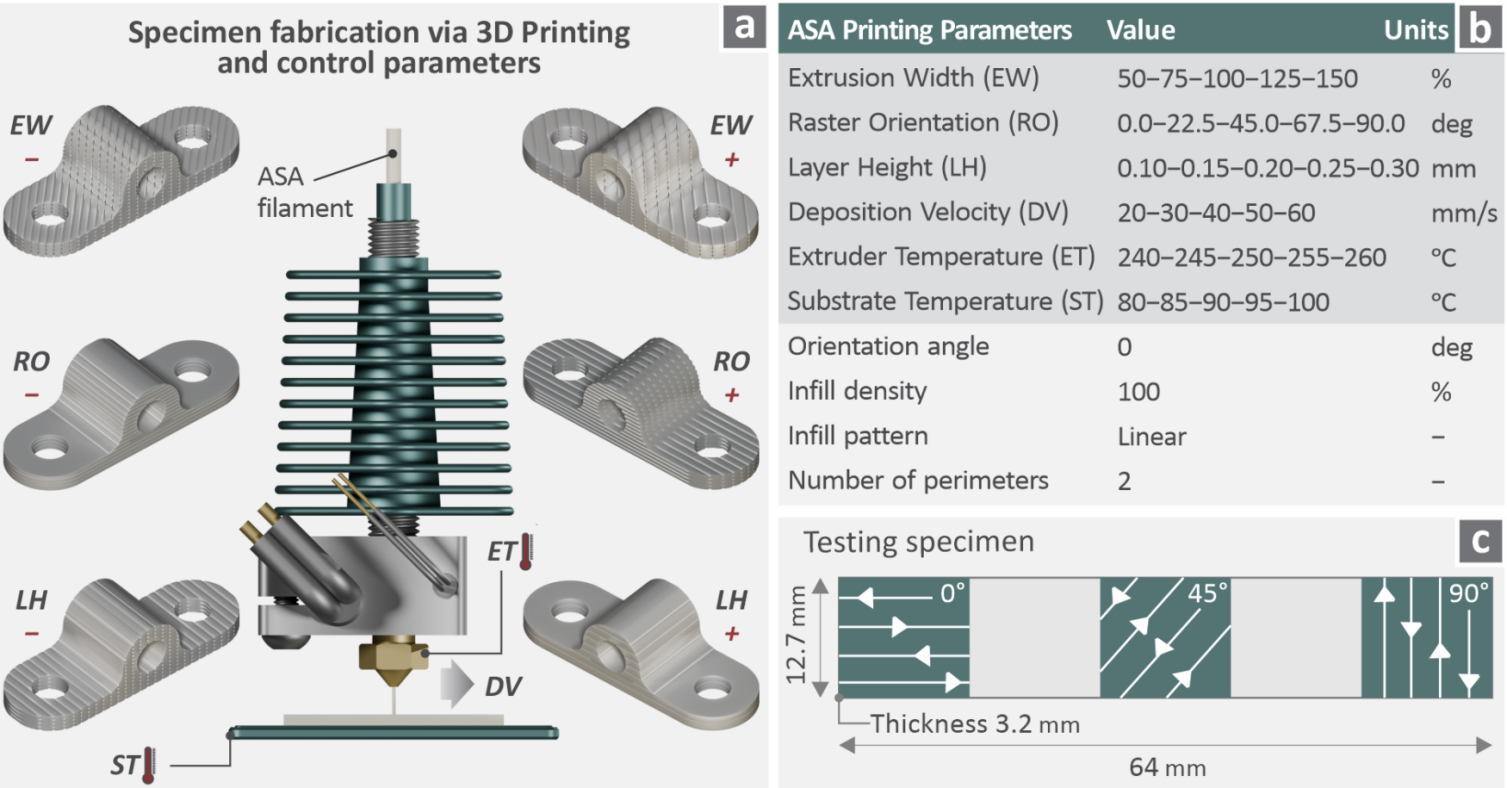
(a) Illustration of control parameters for specimen fabrication with the 3D printing procedure, (b) ASA printing parameters with their levels, and (c) specimen geometry and infill structure.

### 2.5 Coupon Morphology Inspection

The morphology of the 3D-printed coupons was examined by subjecting their lateral and fractured surfaces to scanning electron microscopy (SEM). The information was obtained using a JSM-6362LV (Jeol Ltd., Peabody, Massachusetts) apparatus on Au-sputtered samples under high vacuum and at 20 kV. The coupons also underwent stereoscopic examination using an optical stereoscope OZR5, equipped with an ODC 832 5MP camera (Kern, Balingen, Germany).

The quality characteristics related to the surface roughness were assessed with a Contour GT (Bruker Nano GmbH, Berlin, Germany) device. The average (*R*_a_) (average of the absolute values of the measured surface deviations, i.e., hills and valleys, measured from the average profile line of the examined length), and the root-mean-square roughness (*R*_q_) (square root of the average of the squares of the surface variations, i.e., hills and valleys, of the surface height measured from the average profile line of the examined length) were measured.

The porosity of the 3D printing structure in the ASA samples and their geometrical accuracy (deviation between the nominal and fabricated geometry) were assessed with microcomputed tomography (μ-CT). A Tomoscope HV Compact 225 kV Micro Focus system (Werth Messtechnik GmbH, Gießen, Germany) was employed for analysis. Geometry accuracy assessment was carried out by adjusting the apparatus at a resolution of 60 L, whereas for the pore examination, the apparatus was adjusted at a resolution of 16 L. The acquired μ-CT data were analyzed using the software platform VG Studio MAX 2.2 (Volume Graphics GmbH, Heidelberg, Germany). The methodology is more analytically presented in the Supporting Information.

### 2.6 Orthogonal Arrays, Taguchi Method, ANOVA, and Regression

As the number of process parameters increases, numerous experiments and simulations must be conducted, which is not feasible to accomplish with a single research effort. Thus, the Taguchi optimization method was utilized. The design of orthogonal arrays is used by the Taguchi method to examine the total number of parameters and minimize the number of experiments required to solve a problem.^127^ The orthogonal array compilation is derived through the utilization of the total degree of freedom (DOF), which is extensively analyzed in the Supporting Information, based on information extracted from related literature.^128–138^

For the purposes of this research, the Taguchi L25 orthogonal array was employed, resulting in 125 response sets, as 25 experiments, with 5 repetitions each, were conducted to ensure statistical reliability and repeatability of the results. Five replicas is the common practice in standards for mechanical testing; therefore, a safe amount of replicas is used. For every specimen, five measurements were taken, and the average value was used as the representative result for that specimen. Then, for each experimental run, the mean value and standard deviation of the five replicas were calculated and reported. This procedure provided sufficient statistical power while limiting the experimental effort. Regarding outliers, no data points were removed; all experimental values were included in the statistical analysis. Six control parameters (the most critical 3D printing settings, as stated in the bibliography for MEX 3D printing^87,100^ ), namely, EW, RO, LH, DV, ET, and ST, were examined and are presented in Table 1 together with their levels. Their selection was based on the existing modeling and optimization bibliography of the MEX 3D printing parameter levels,^87,100^ as well as preliminary screening tests.

**Table 1:** Taguchi L25: Control Parameters and Their Levels

Run	EW (%)	RO (deg)	LH (mm)	DV (mm/s)	ET (°C)	ST (°C)
1	50	0.0	0.10	20	240	80
2	50	22.5	0.15	30	245	85
3	50	45.0	0.20	40	250	90
4	50	67.5	0.25	50	255	95
5	50	90.0	0.30	60	260	100
6	75	0.0	0.15	40	255	100
7	75	22.5	0.20	50	260	80
8	75	45.0	0.25	60	240	85
9	75	67.5	0.30	20	245	90
10	75	90.0	0.10	30	250	95
11	100	0.0	0.20	60	245	95
12	100	22.5	0.25	20	250	100
13	100	45.0	0.30	30	255	80
14	100	67.5	0.10	40	260	85
15	100	90.0	0.15	50	240	90
16	125	0.0	0.25	30	260	90
17	125	22.5	0.30	40	240	95
18	125	45.0	0.10	50	245	100
19	125	67.5	0.15	60	250	80
20	125	90.0	0.20	20	255	85
21	150	0.0	0.30	50	250	85
22	150	22.5	0.10	60	255	90
23	150	45.0	0.15	20	260	95
24	150	67.5	0.20	30	240	100
25	150	90.0	0.25	40	245	80

The control parameters were selected, as mentioned above, by reviewing the related literature on the impact of 3D printing parameters on the performance of parts built using the MEX 3D printing method. The most generic parameters and those proven in the literature to be the most critical for the performance of the 3D-printed parts were selected for study. The literature review did not reveal any similar publications for 3D-printed ASA parts that could be used for this selection. Furthermore, most studies examined a limited number of parameters simultaneously. Herein, six 3D printing settings were simultaneously examined to reveal possible synergistic relationships between them and identify a set of parameter values that would yield better results in the quality metrics. The levels of parameters were determined by studying the respective literature on 3D-printed ASA MEX parts. Furthermore, preliminary prints were conducted to locate the range of values that did not encounter printability issues. Specifically for the lower and upper values for parameters, the material datasheet was also consulted for specific parameters, such as the extrusion temperature.

ANOVA was used to evaluate the statistically significant differences in each parameter through interpretation of the experimental outcome.^139^ The theory employed is presented in detail in the Supporting Information. Moreover, the theory and formulas for the implementation of the three regression models employed, i.e., RQRM, LRM, and QRM, can also be found in the Supporting Information. Their application aimed to compile models for each output as functions of the control parameters. LRM is a first-degree equation, while RQRM and QRM are more advanced. Their comparison was necessary for deriving the simplest equation, which could be utilized to maintain a reliable and beneficial prediction outcome. Two additional confirmation runs verified the precision of the predictive models.

## 3 Results

### 3.1 Structural and Morphological Characterization

Figure 4a presents the void compactness, sphericity, and diameter graphs for Runs 1, 7, 13, 19, and 25, which belong to the diagonal of the area with the total number of runs (these were selected to be investigated as more characteristic for further analysis, as they include the entire parameters’ range assessed herein). These were accompanied by the values of the 3D printing parameters. Run 7 had the highest number of voids (18,870). Figure 4b shows a 3D view of the void analysis for the ASA Run 3 sample through color coding mapping after conducting a CT scan of 16 L, while Figure 4c shows the *P*_CT_ levels in bars for all of the experimental runs. It is remarkable that the levels of Runs 18, 21, and 22 were much lower than those of the rest of the runs. The significant differences in the porosity of the samples from different runs highlight the importance of identifying and utilizing proper 3D printing parameter sets. This by itself can notably improve the porosity of the 3D printing structure and, as a result, the integrity and robustness of the fabricated parts.

**Figure 4:**
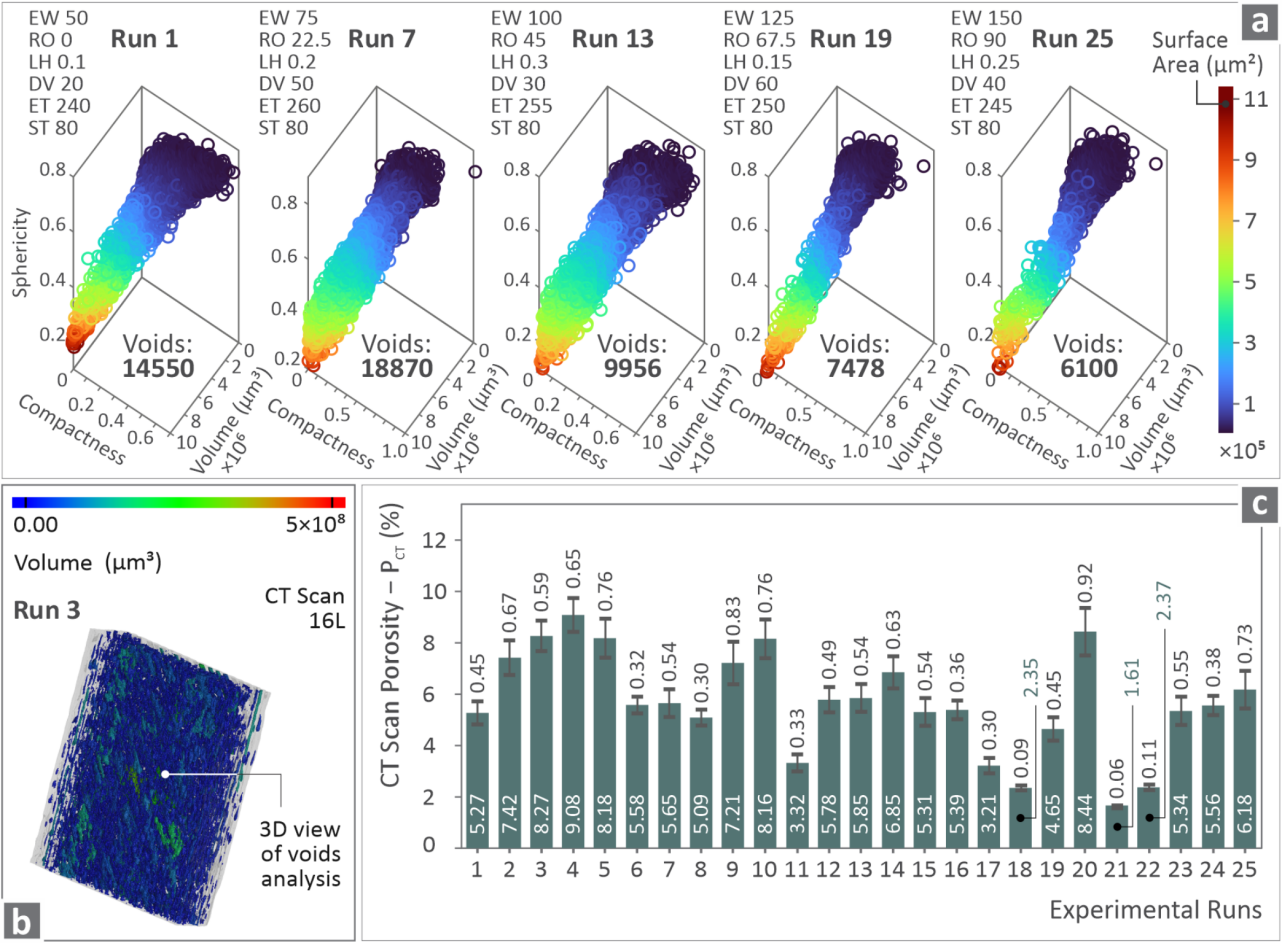
(a) Void-derived data with regard to Runs 1, 7, 13, 19, and 25 (belonging to the diagonal), (b) color coding mapping on ASA Run 3 sample indicating its volume, and (c) bars of *P*_CT_ for each of the experimental runs.

Figure 5a shows histograms and their integrals for the runs of the diagonal (1, 7, 13, 19, 25), showing the relative surface and deviating points vs the actual-to-nominal dimensional deviation. In Figure 5b, color coding mapping was utilized again to present an ASA sample 3D view of the dimensional deviation analysis after a CT scan of 60 L resolution. Figure 5c shows the bars of A2N_95_ for all experimental runs. Again, significant differences in the geometrical accuracy of the samples from different runs can be observed, similar to the porosity findings. These are discussed and analyzed in the following.

**Figure 5:**
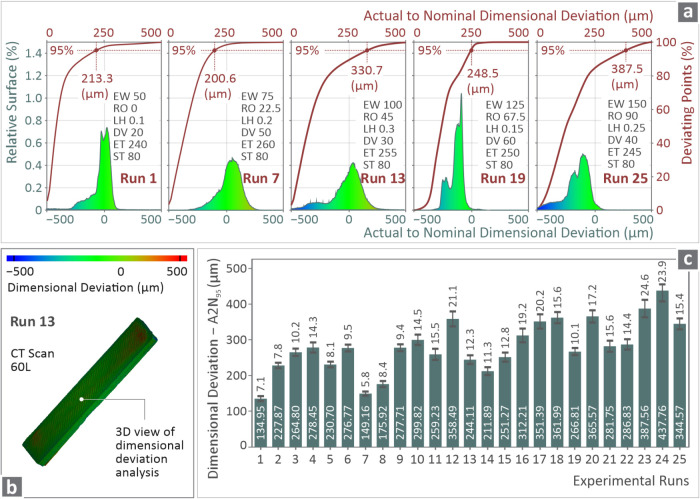
(a) Histograms and their integrals for the diagonal runs in relative surface and deviating points vs actual-to-nominal graphs for geometry deviations, (b) 3D view of the dimensional deviation for Run 13 ASA sample through color coding mapping, and (c) A2N_95_ bars for all of the experimental runs.

Figure 6 presents the optical-profile-related information, including an image captured during the examination (Figure 6a) and a roughness map of a Run 22 ASA sample (Figure 6b). Figure 6c and d shows the levels of roughness *R*_a_ and *R*_q_ in the bars for all experimental runs.

**Figure 6:**
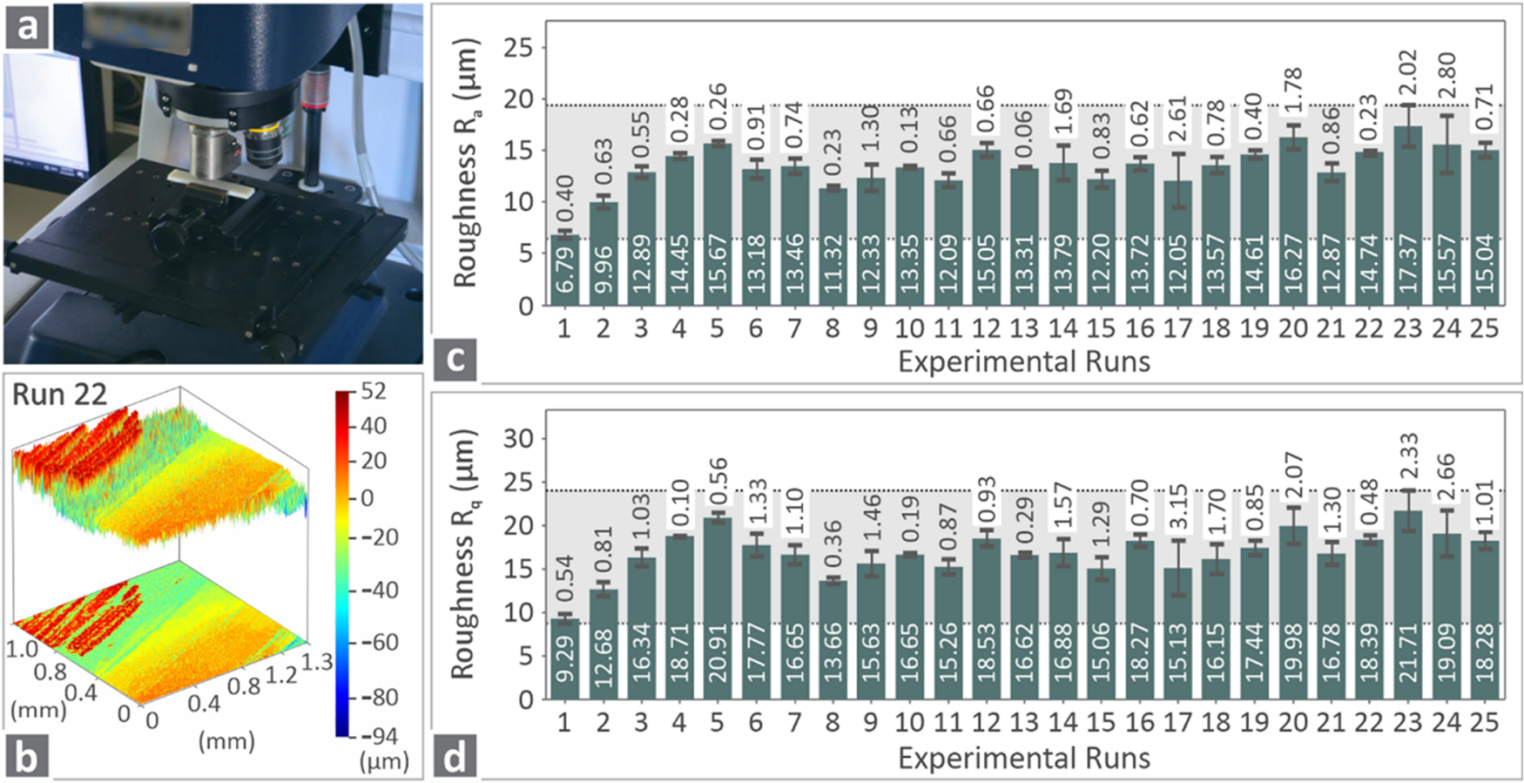
(a) Image captured during the optical profilometry of a random specimen, (b) roughness map of a Run 22 sample, and (c, d) roughness *R*_a_ and *R*_q_ bars for all of the experimental runs.

Figure 7 shows the stereoscopic images captured from the total number of runs (1–25), accompanied by the respective printing parameter values (EW, RO, LH, DV, E, and ST). The runs belonging to the diagonal are highlighted. The magnification used for the images was 5×. Differences in the 3D printing structure due to variations in the 3D printing settings (sets of parameter values) across the runs are visible in the illustrations.

**Figure 7:**
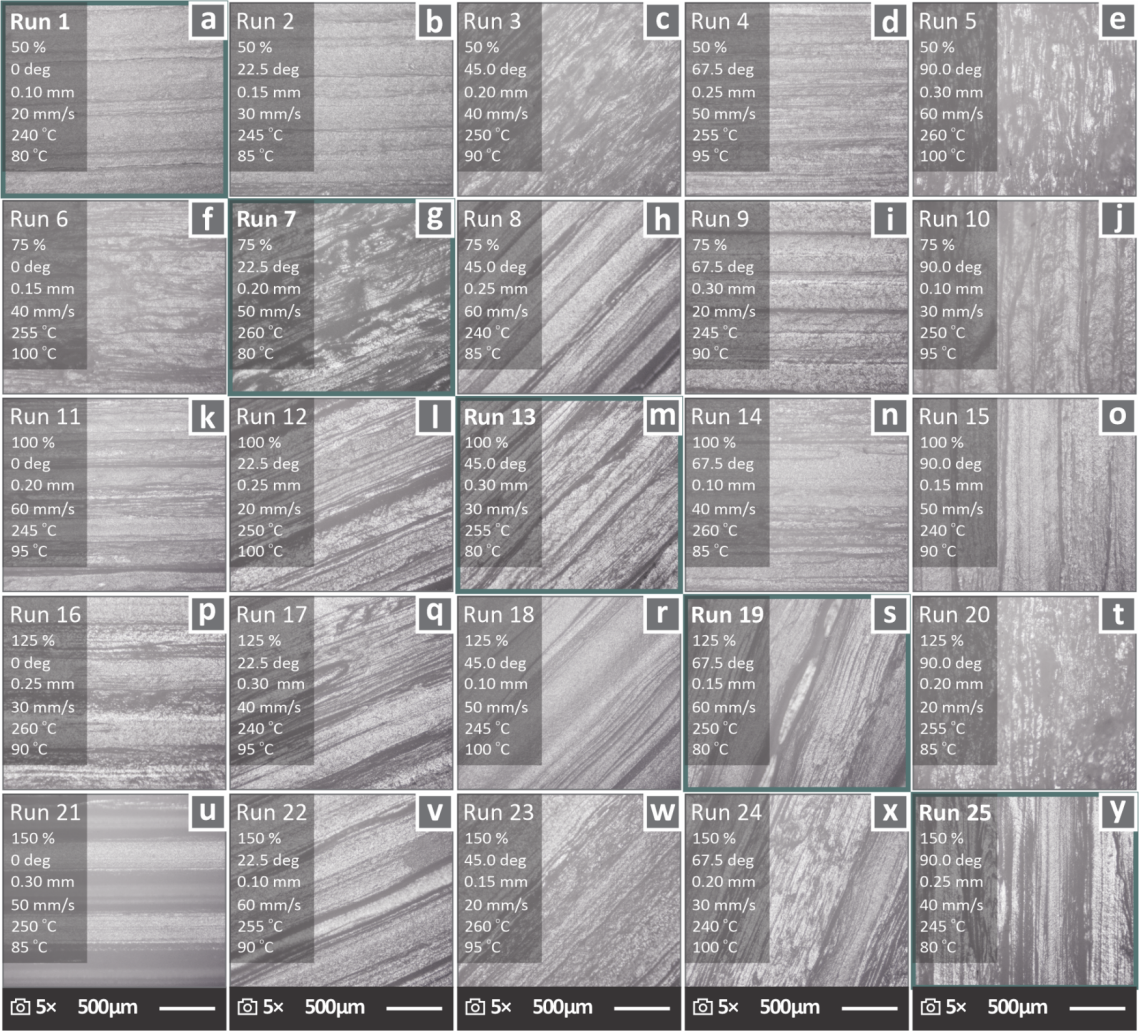
Stereoscopic images of ASA specimens belonging to each of the 25 experimental runs, in 5× magnifications, accompanied by the values of their 3D printing parameters, namely, EW, RO, LH, DV, E, and ST.

### 3.2 Experimental Data

The control parameter EW, RO, LH, DV, E, and ST rankings for means *R*_a_, *R*_q_, A2N_95_, and *P*_CT_ are listed in Table 2 based on the delta statistics. The calculation procedure followed for all of the factors included calculating the deviation by subtracting the smallest average value from the highest. The impact of the delta value at the highest level (rank 1) on the response metric was significant, whereas the delta value at the lowest level (rank 6) did not have a considerable impact on the response metric. Control parameters EW and ET were ranked higher, as presented in Table 2, specifically, EW in the case of A2N_95_ and *P*_CT_, while ET in the case of *R*_a_ and *R*_q_. The lower-ranked parameters were DV for *R*_a_ and *R*_q_, ET for A2N_95_, and ST for *P*_CT_.

**Table 2:** Control Parameter Ranking for the Average Responses (R_a_, *R*_q_, A2N_95_, *P*_CT_)

Level	EW (%)	RO (deg)	LH (mm)	DV (mm/s)	ET (°C)	ST (°C)
*R*_a_ (μm)						
1	11.95	11.73	12.45	13.56	11.59	12.64
2	12.73	13.05	13.46	13.18	12.6	12.84
3	13.29	13.69	14.06	13.39	13.76	13.17
4	14.04	14.15	13.91	13.31	14.39	13.86
5	15.12	14.51	13.25	13.68	14.8	14.61
Delta	3.16	2.78	1.61	0.5	3.22	1.97
Rank	2	3	5	6	1	4
*R*_q_ (μm)						
1	15.59	15.47	15.47	17.03	14.44	15.65
2	16.07	16.27	16.93	16.66	15.6	16
3	16.47	16.89	17.46	16.88	17.15	16.74
4	17.39	17.55	17.49	16.67	18.29	17.49
5	18.85	18.17	17.01	17.13	18.88	18.49
Delta	3.26	2.7	2.02	0.47	4.44	2.83
Rank	2	4	5	6	1	3
A2N95 (μm)						
1	227.4	253	259.1	304.9	270.3	227.9
2	235.9	274.8	282.1	304.4	294.3	252.6
3	265	286.9	295.3	289.9	294.3	278.6
4	331.6	294.5	293.9	264.5	290.3	315.3
5	347.7	298.4	277.1	243.9	258.3	333.1
Delta	120.3	45.4	36.2	61	36	105.2
Rank	1	4	5	3	6	2
PCT (%)						
1	7.646	4.234	4.999	6.41	4.888	5.518
2	6.337	4.887	5.661	6.476	5.296	5.882
3	5.423	5.381	6.246	6.018	5.692	5.71
4	4.807	6.671	6.304	4.8	6.264	5.824
5	4.212	7.252	5.214	4.721	6.284	5.49
Delta	3.434	3.018	1.305	1.755	1.396	0.392
Rank	1	2	5	3	4	6

Apart from the ranking, for every response metric, in Table 2, the average value calculated for each control parameter level is depicted. The overall mean and standard deviation values for the *R*_a_, *R*_q_, A2N_95_, and *P*_CT_ responses (25 runs) are depicted in Table 3. Table S1 in the Supporting Information contains additional information and data for all 25 experimental runs.

**Table 3:** Average and Standard Deviations Values of the Experimental Findings for the Responses (*R*_a_, *R*_q_, A2N_95_, *P*_CT_)

Run	*R*_a_ (μm)	*R*_q_ (μm)	A2N95 (μm)	PCT (%)
1	6.79 ± 0.40	9.29 ± 0.54	134.95 ± 7.10	5.27 ± 0.45
2	9.96 ± 0.63	12.68 ± 0.81	227.87 ± 7.75	7.42 ± 0.67
3	12.89 ± 0.55	16.34 ± 1.03	264.80 ± 10.18	8.27 ± 0.59
4	14.45 ± 0.28	18.72 ± 0.10	278.45 ± 14.29	9.08 ± 0.65
5	15.67 ± 0.26	20.91 ± 0.56	230.70 ± 8.08	8.18 ± 0.76
6	13.18 ± 0.91	17.77 ± 1.32	276.77 ± 9.47	5.58 ± 0.32
7	13.46 ± 0.74	16.65 ± 1.10	149.16 ± 5.80	5.65 ± 0.54
8	11.32 ± 0.23	13.66 ± 0.36	175.92 ± 8.44	5.09 ± 0.31
9	12.33 ± 1.30	15.63 ± 1.46	277.71 ± 9.45	7.21 ± 0.83
10	13.35 ± 0.13	16.65 ± 0.19	299.82 ± 14.47	8.16 ± 0.76
11	12.09 ± 0.66	15.26 ± 0.87	259.23 ± 15.48	3.32 ± 0.33
12	15.05 ± 0.66	18.53 ± 0.93	358.49 ± 21.09	5.78 ± 0.49
13	13.31 ± 0.06	16.62 ± 0.29	244.11 ± 12.34	5.85 ± 0.54
14	13.79 ± 1.69	16.88 ± 1.57	211.89 ± 11.32	6.85 ± 0.63
15	12.20 ± 0.83	15.06 ± 1.29	251.27 ± 12.80	5.31 ± 0.54
16	13.72 ± 0.62	18.27 ± 0.70	312.21 ± 19.21	5.39 ± 0.36
17	12.05 ± 2.61	15.13 ± 3.15	351.39 ± 20.19	3.21 ± 0.30
18	13.57 ± 0.78	16.15 ± 1.70	361.99 ± 15.61	2.35 ± 0.09
19	14.61 ± 0.40	17.44 ± 0.85	266.81 ± 10.09	4.65 ± 0.45
20	16.27 ± 1.18	19.98 ± 2.07	365.57 ± 17.20	8.44 ± 0.92
21	12.87 ± 0.86	16.78 ± 1.30	281.75 ± 15.56	1.61 ± 0.06
22	14.74 ± 0.23	18.39 ± 0.48	286.83 ± 14.44	2.37 ± 0.11
23	17.37 ± 2.02	21.71 ± 2.33	387.56 ± 24.62	5.35 ± 0.55
24	15.57 ± 2.80	19.09 ± 2.66	437.76 ± 23.93	5.56 ± 0.38
25	15.04 ± 0.71	18.27 ± 1.01	344.57 ± 15.36	6.18 ± 0.73

### 3.3 ANOVA Resulting Data and Respective Regression Model Equations

As mentioned above, three regression models were implemented. The RQRM is presented more analytically, as the analysis revealed that it is the most efficient among the three. This is presented in the following. Tables 4–7 present the polynomial ANOVA results, indicating the RQRM values for *R*_a_, *R*_q_, A2N_95_, and *P*_CT_, respectively, vs EW, RO, LH, DV, ET, and ST. Each table is accompanied by the equation compiled for the calculation of the respective response metric (eqs 1–4).

1 $$R_{a}=-319+0.0123\times EW+0.0564\times RO+51.01\times LH-0.0660\times DV+2.63\times ET-0.643\times ST+0.0000913\times EW^{2}-0.000298\times RO^{2}-117.3\times LH^{2}+0.000872\times DV^{2}-0.00493\times ET^{2}+0.00412\times ST^{2}$$

2 $$R_{q}=-305-0.0250\times EW+0.0338\times RO+57.3\times LH-0.0679\times DV+2.43\times ET-0.538\times ST+0.000282\times EW^{2}-0.000045\times RO^{2}-125.1\times LH^{2}+0.000876\times DV^{2}-0.00439\times ET^{2}+0.00378\times ST^{2}$$

3 $$A2N95=-21074+0.143\times EW+1.003\times RO+1172\times LH+1.305\times DV+165.4\times ET+6.95\times ST+0.00601\times EW^{2}-0.00568\times RO^{2}-2690\times LH^{2}-0.0365\times DV^{2}-0.3319\times ET^{2}-0.0083\times ST^{2}$$

4 $$PCT=-145.6-0.0730\times EW+0.02649\times RO+48.21\times LH+0.0094\times DV+0.935\times ET+0.568\times ST+0.0001973\times EW^{2}+0.0000918\times RO^{2}-115.2\times LH^{2}-0.000749\times DV^{2}-0.00172\times ET^{2}-0.00317\times ST^{2}$$

**Table 4:** Polynomial ANOVA: RQRM Values for the Response *R*_a_ vs EW, RO, LH, DV, ET, and ST

Source	DF	Adj. SS	Adj. MS	*F*-value	*P*-value
Regression	12	548.688	45.7240	40.00	0.000
EW	1	0.507	0.5069	0.44	0.507
RO	1	32.364	32.3636	28.31	0.000
LH	1	34.813	34.8127	30.45	0.000
DV	1	2.333	2.3329	2.04	0.156
ET	1	6.059	6.0589	5.30	0.023
ST	1	2.787	2.7872	2.44	0.121
EW^2^	1	1.139	1.1393	1.00	0.320
RO^2^	1	7.961	7.9610	6.96	0.010
LH^2^	1	30.087	30.0870	26.32	0.000
DV^2^	1	2.661	2.6608	2.33	0.130
ET^2^	1	5.326	5.3262	4.66	0.033
ST^2^	1	3.717	3.7169	3.25	0.074
Error	112	128.031	1.1431		
Total	124				
*R* ^2^	81.08%				
*R* ^2^ (adj.)	79.05%				
*R* ^2^ (pred.)	76.41%				

**5:** Polynomial ANOVA: RQRM Values for the Response *R*_q_ vs EW, RO, LH, DV, ET, and ST

Source	DF	Adj. SS	Adj. MS	*F*-value	*P*-value
Regression	12	817.120	68.0933	39.17	0.000
EW	1	2.091	2.0915	1.20	0.275
RO	1	11.613	11.6133	6.68	0.011
LH	1	43.990	43.9901	25.31	0.000
DV	1	2.469	2.4692	1.42	0.236
ET	1	5.161	5.1606	2.97	0.088
ST	1	1.952	1.9525	1.12	0.292
EW^2^	1	10.874	10.8740	6.26	0.014
RO^2^	1	0.184	0.1838	0.11	0.746
LH^2^	1	34.252	34.2522	19.70	0.000
DV^2^	1	2.685	2.6855	1.54	0.216
ET^2^	1	4.225	4.2247	2.43	0.122
ST^2^	1	3.133	3.1333	1.80	0.182
Error	112	194.697	1.7384		
Total	124				
*R* ^2^	80.76%				
*R* ^2^ (adj.)	78.70%				
*R* ^2^ (pred.)	76.10%				

**6:** Polynomial ANOVA: RQRM Values for the Response A2N_95_ vs EW, RO, LH, DV, ET, and ST

Source	DF	Adj. SS	Adj. MS	*F*-value	*P*-value
Regression	12	625,534	52127.8	140.62	0.000
EW	1	68	68.2	0.18	0.669
RO	1	10,246	10246.1	27.64	0.000
LH	1	18,371	18370.7	49.56	0.000
DV	1	911	911.1	2.46	0.120
ET	1	23,931	23930.8	64.55	0.000
ST	1	326	325.8	0.88	0.351
EW^2^	1	4945	4944.9	13.34	0.000
RO^2^	1	2898	2898.3	7.82	0.006
LH^2^	1	15,826	15826.1	42.69	0.000
DV^2^	1	4670	4670.0	12.60	0.001
ET^2^	1	24,096	24095.6	65.00	0.000
ST^2^	1	15	14.9	0.04	0.841
Error	112	41,519	370.7		
Total	124				
*R* ^2^	93.78%				
*R* ^2^ (adj.)	93.11%				
*R* ^2^ (pred.)	92.30%				

**Table 7:** Polynomial ANOVA: RQRM Values for the Response *P*_CT_ vs EW, RO, LH, DV, ET, and ST

Source	DF	Adj. SS	Adj. MS	*F*-value	*P*-value
Regression	12	471.182	39.2652	95.36	0.000
EW	1	17.844	17.8441	43.34	0.000
RO	1	7.146	7.1456	17.35	0.000
LH	1	31.097	31.0967	75.52	0.000
DV	1	0.047	0.0474	0.12	0.735
ET	1	0.764	0.7642	1.86	0.176
ST	1	2.179	2.1785	5.29	0.023
EW^2^	1	5.320	5.3198	12.92	0.000
RO^2^	1	0.756	0.7563	1.84	0.178
LH^2^	1	29.007	29.0074	70.45	0.000
DV^2^	1	1.966	1.9658	4.77	0.031
ET^2^	1	0.646	0.6463	1.57	0.213
ST^2^	1	2.199	2.1986	5.34	0.023
Error	112	46.115	0.4117		
Total	124				
*R* ^2^	91.09%				
*R* ^2^ (adj.)	90.13%				
*R* ^2^ (pred.)	88.90%				

From the three regression models implemented, a table was formed to compare their efficiency. Table 8 presents a comparison of the three existing regression models, namely, LRM, RQRM, and QRM. The RQRM- and QRM-derived data for the total number of response metrics were noticeably better than the data from LRM. Still, the RQRM results were sufficient enough to be chosen over the more complex QRM, and consequently, eqs 5–8 (LRM) and 9–12 (QRM) were not further utilized in the research.

**Table 8:** Comparison between LRM, RQRM, and QRM

	LRM			RQRM			QRM		
	*R* ^2^ (%)	*R* ^2^ (adj., %)	*F*-value	*R* ^2^ (%)	*R* ^2^ (adj., %)	*F*-value	*R* ^2^ (%)	*R* ^2^ (adj., %)	*F*-value
*R*_a_ (μm)	73.56	72.22	54.72	81.08	79.05	40.00	81.36	78.40	27.48
*R*_q_ (μm)	75.29	74.03	59.91	80.76	78.70	39.17	81.01	77.99	26.84
A2N95 (μm)	85.91	85.20	119.94	93.78	93.11	140.62	94.95	94.15	118.34
PCT (%)	83.37	82.53	98.62	91.09	90.13	95.36	91.98	90.70	72.14

Tables 9 and 10 present the model comparison for all responses. LRM, RQRM, and QRM were evaluated using adjusted *R* ^2^, predictive *R* ^2^, PRESS, AICc, and BIC. Model selection was based primarily on ΔAICc, with PRESS and ΔBIC used as supporting criteria. RQRM was retained for *R*_a_, *R*_q_, and PCT, while QRM was retained for A2N95.

**Table 9:** Model Selection Criteria for *R*_a_ and *R*_q_

Response	*R*_a_			*R*_q_		
Model	LRM	RQRM	QRM	LRM	RQRM	QRM
# parameters	7	13	18	7	13	18
*R* ^2^(adj.)	72.22%	79.05%	78.40%	74.03%	78.70%	77.99%
*R* ^2^(pred.)	70.33%	76.41%	74.25%	72.37%	76.10%	73.85%
PRESS	200.761	159.619	174.283	279.599	241.871	264.568
AICc	416.81	389.55	401.08	458.64	441.94	453.74
ΔAICc	27.26	0.00	11.53	16.70	0.00	11.80
BIC	438.19	425.33	447.58	480.03	477.72	500.24
ΔBIC	12.86	0.00	22.25	2.31	0.00	22.52
Decision	Not retained	**Retained**	Not retained	Not retained	**Retained**	Not retained

**Table 10:** Model Selection Criteria for A2N95 and PCT

Response	A2N95			PCT		
Model	LRM	RQRM	QRM	LRM	RQRM	QRM
# parameters	7	13	18	7	13	18
*R* ^2^(adj.)	85.20%	93.11%	94.15%	82.53%	90.13%	90.70%
*R* ^2^(pred.)	84.27%	92.30%	93.09%	81.36%	88.90%	89.04%
PRESS	104940	51390	46098.6	96.4052	57.4117	56.7137
AICc	1199.78	1112.25	1099.54	325.24	261.91	262.18
ΔAICc	100.24	12.71	0.00	63.33	0.00	0.27
BIC	1221.16	1148.03	1146.04	346.63	297.68	308.68
ΔBIC	75.12	1.99	0.00	48.95	0.00	11.00
Decision	Not retained	Not retained	**Retained**	Not retained	**Retained**	Not retained

The comparison of alternative regression models (Tables 9 and 10) shows that the reduced quadratic form is sufficient to describe most responses. For *R*_a_ and *R*_q_, RQRM is strongly preferred (ΔAICc ≈ 11–12, lower PRESS, higher *R* ^2^ pred.). For A2N95, the QRM is retained (ΔAICc ≈ 12.7, lower PRESS). For PCT, the two models are essentially equivalent in AICc (ΔAICc = 0.27), but the substantially lower BIC favors the RQRM, which we therefore retain. For *R*_a_, *R*_q_, and PCT, the RQRM achieved lower AICc and PRESS values while maintaining a strong predictive *R* ^2^, indicating that the additional terms in the full quadratic model did not improve predictive ability. In contrast, for A2N95, the full quadratic model provided a clear advantage, with markedly lower PRESS and the lowest AICc, and was therefore retained. Overall, these results underline that quadratic terms can be useful, but including all possible higher-order effects is not always necessary. These criteria confirm that RQRM achieves comparable or superior predictive performance while avoiding unnecessary model complexity.

The linear regression model equations for *R*_a_, *R*_q_, A2N95, and PCT are as follows:

5 $$R_{a}=-41.95+0.03057\times EW+0.02956\times RO+4.10\times LH+0.00373\times DV+0.1644\times ET+0.0991\times ST$$

6 $$R_{q}=-59.88+0.03140\times EW+0.02970\times RO+7.29\times LH+0.00215\times DV+0.2314\times ET+0.1432\times ST$$

7 A2N95=−182.1+1.3456×EW+0.4915×RO+95.9×LH−1.617×DV−0.557×ET+5.463×ST

8 PCT=−9.52−0.03359×EW+0.03475×RO+2.15×LH−0.05055×DV+0.0752×ET−0.0023×ST

The quadratic regression model equations for *R*_a_, *R*_q_, A2N95, and PCT are as follows:

9 $$R_{a}=210-0.481\times EW+0.0952\times RO+47.8\times LH-0.167\times DV-1.33\times ET-1.14\times ST+0.000126\times EW^{2}-0.000580\times RO^{2}-149.1\times LH^{2}+0.00136\times DV^{2}+0.00281\times ET^{2}+0.00737\times ST^{2}+0.000210\times EW\times RO+0.218\times EW\times LH+0.00112\times EW\times DV+0.00154\times EW\times ET-0.000448\times RO\times DV$$

10 $$R_{q}=466-0.841\times EW+0.0780\times RO+65.5\times LH-0.162\times DV-3.21\times ET-1.52\times ST+0.000324\times EW^{2}-0.000344\times RO^{2}-180.8\times LH^{2}+0.00075\times DV^{2}+0.0065\times ET^{2}+0.0099\times ST^{2}+0.000278\times EW\times RO+0.233\times EW\times LH+0.00168\times EW\times DV+0.00271\times EW\times ET-0.000544\times RO\times DV$$

11 $$A2N95=-70506+48.9\times EW-3.330\times RO-460\times LH+2.18\times DV+506.4\times ET+125.4\times ST+0.00265\times EW^{2}+0.00445\times RO^{2}+2384\times LH^{2}+0.0221\times DV^{2}-0.985\times ET^{2}-0.695\times ST^{2}+0.00475\times EW\times RO-8.10\times EW\times LH-0.0897\times EW\times DV-0.1717\times EW\times ET+0.04364\times RO\times DV$$

12 $$PCT=-320+0.207\times EW-0.0136\times RO+14.6\times LH-0.1506\times DV+1.54\times ET+2.442\times ST+0.000176\times EW^{2}-0.000112\times RO^{2}-63.9\times LH^{2}+0.001166\times DV^{2}-0.00258\times ET^{2}-0.01340\times ST^{2}+0.000485\times EW\times RO+0.144\times EW\times LH+0.000090\times EW\times DV-0.00131\times EW\times ET+0.000271\times RO\times DV$$

In Figure 8, the main effect plots reveal the effects of control factors EW, RO, LH, DV, ET, and ST on response metrics *R*_a_, *R*_q_, A2N_95_, and *P*_CT_. Figure 8a shows *R*_a_ (left side) and *R*_q_ (right side), while Figure 8b shows A2N_95_ (left side) and *P*_CT_ (right side). Figure 8a,b also shows tensile strength results (right side) derived from previously conducted work, for completeness and to reveal possible correlations between the findings.^100^ As observed, the ET control parameter had the greatest effect on *R*_a_ and *R*_q_, whereas EW significantly influenced A2N_95_ and *P*_CT_. Regarding tensile strength, the most influential parameter was RO. The behaviors of *R*_a_ and *R*_q_ seem to be remarkably similar when variying all control parameters, whereas the same is true for A2N_95_ and *P*_CT_ with LH and DV. The remaining control parameters influence the two responses differently (RO and ET) or vice versa (EW, ST). It should be noted that for all of the responses herein, the smaller the better criterion is preferred, as smaller values indicate better quality characteristics. This is not the case for tensile strength, where the larger the better criterion is preferable. The curves should be evaluated, taking into account these aspects of the responses. These are analyzed as follows.

**Figure 8:**
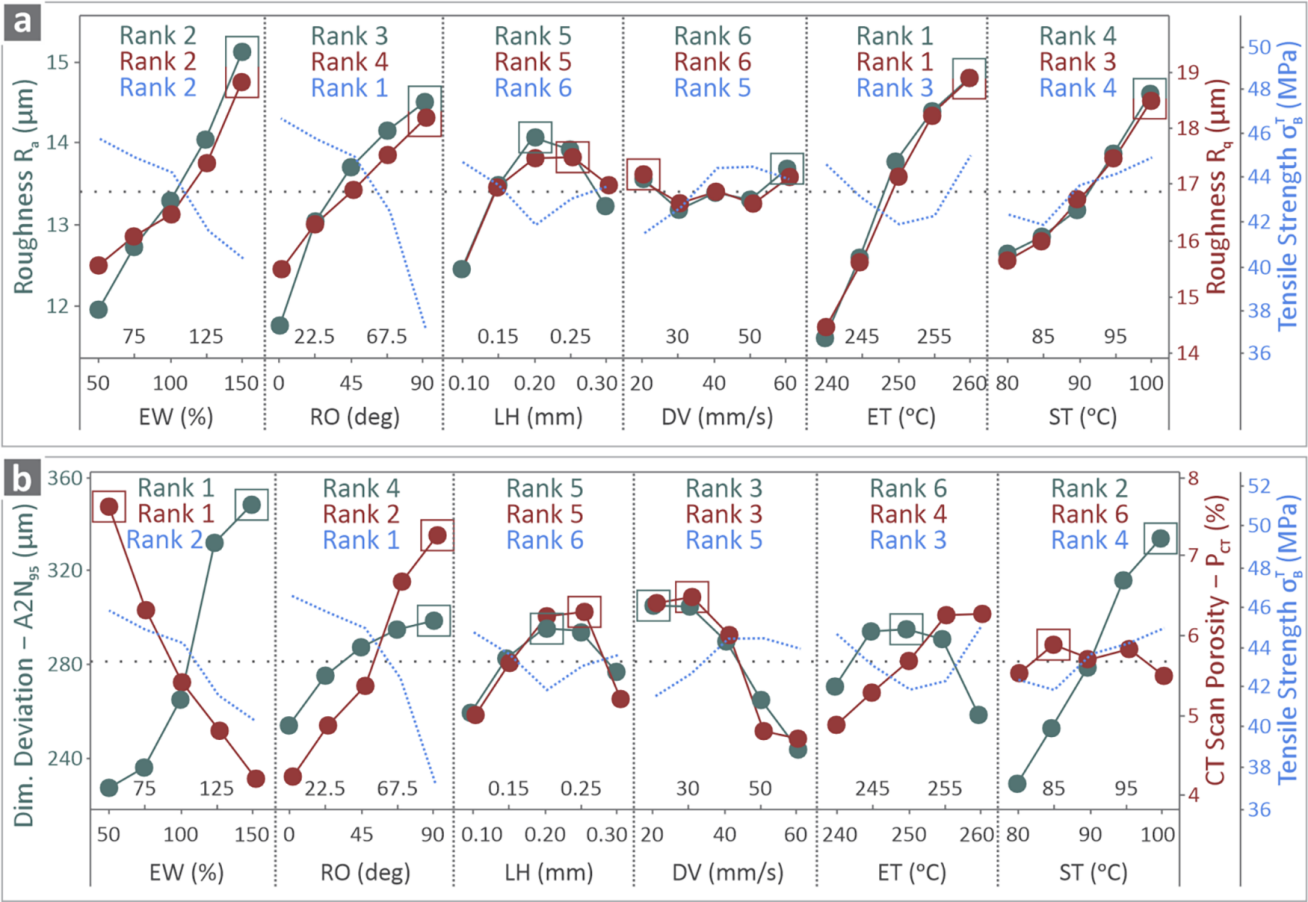
Main effect plots of (a) *R*_a_, *R*_q_, and tensile strength and (b) A2N_95_, *P*_CT_, and tensile strength vs EW, RO, LH, DV, ET, and ST, along with their ranks.

### 3.4 Confirmation and Validation Data

Two additional confirmation experiments were conducted (Runs 26 and 27) to validate the prediction functions. Tables 11 and 12 present the control parameter levels for the confirmation runs and the average and standard deviation values derived, respectively. Table S2 of the Supporting Information present experimental results for all replicas of the confirmation runs. Table 13 provide validation data calculated when comparing the experimentally derived and calculated values for the two confirmation runs, accompanied by the calculation of the absolute error (the variation between predicted and experimental values), which was low (less than 10%) for all responses, revealing the reliability of the modeling process outcome. No indication of model bias or heteroscedasticity was observed.

**Table 11:** Confirmation Run Control Parameter Levels

Run	EW (%)	RO (deg)	LH (mm)	DV (mm/s)	ET (°C)	ST (°C)
26	84	31	0.14	30	246	86
27	125	75	0.23	49	256	94

**Table 12:** Mean and Standard Deviations of the Experimental Findings in the Confirmation Runs for the Responses (*R*_a_, *R*_q_, A2N_95_, *P*_CT_)

Run	*R*_a_ (μm)	*R*_q_ (μm)	A2N95 (μm)	PCT (%)
26	12.30 ± 0.44	15.29 ± 0.52	244.23 ± 8.24	5.50 ± 0.18
27	15.80 ± 0.54	19.44 ± 0.56	327.83 ± 10.28	7.21 ± 0.25

**Table 13:** Comparison Table

Run		26	27
Experimental	*R*_a_ (μm)	12.30 ± 0.44	15.80 ± 0.54
	*R*_q_ (μm)	15.29 ± 0.52	19.44 ± 0.56
	A2N95 (μm)	244.23 ± 8.24	327.83 ± 10.28
	PCT (%)	5.50 ± 0.18	7.21 ± 0.25
Predicted	*R*_a_ (μm)	11.60	17.05
	*R*_q_ (μm)	14.02	21.11
	A2N95 (μm)	263.16	355.59
	PCT (%)	5.99	6.54
Absolute error	*R*_a_ (%)	5.63 ± 3.36	8.04 ± 3.69
	*R*_q_ (%)	8.24 ± 3.11	8.64 ± 3.15
	A2N95 (%)	7.85 ± 3.65	8.55 ± 3.39
	PCT (%)	9.11 ± 3.53	9.24 ± 3.20

Figure 9 shows the graphs of the predicted vs experimental values of the *R*_q_ (Figure 9a), A2N_95_ (Figure 9b), and *P*_CT_ (Figure 9c) responses for all 25 experimental runs and two confirmation runs. The predicted and experimentally measured values were remarkably close, especially in the cases of *R*_q_ and A2N_95_.

**Figure 9:**
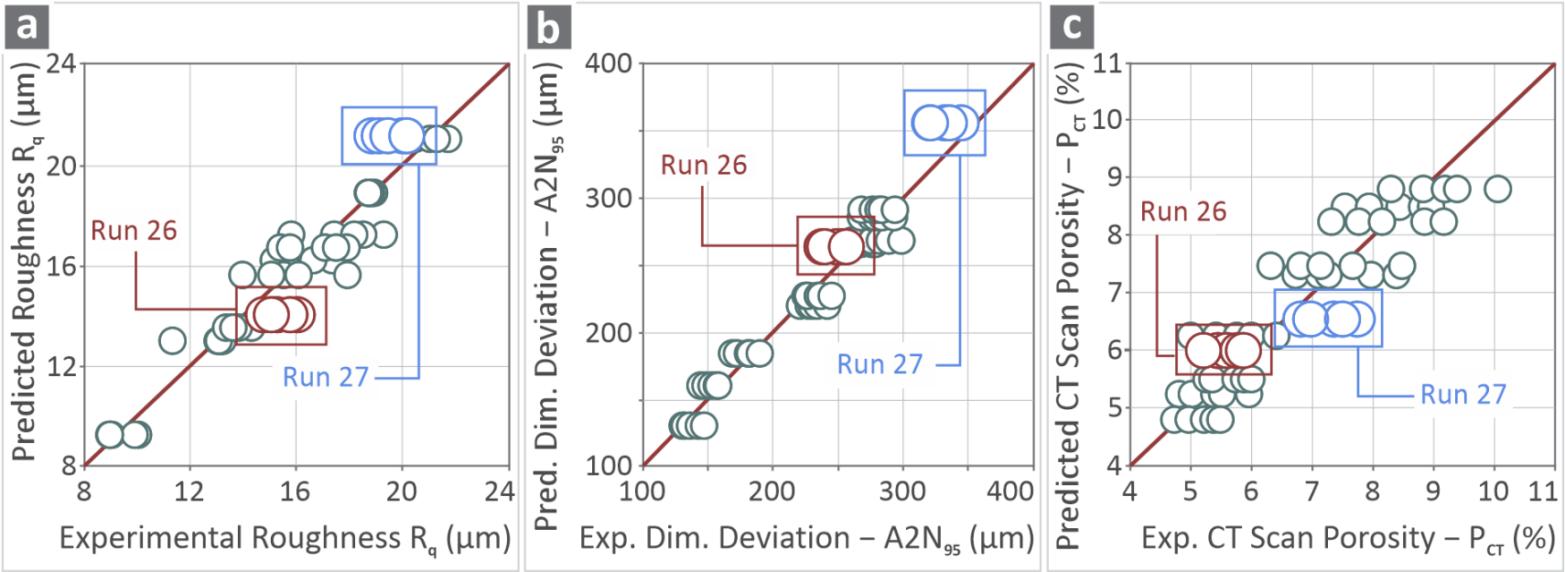
Predicted and experimentally measured values in graphs for (a) *R*_q_, (b) A2N_95_, and (c) *P*_CT_ about the 25 experimental runs and two confirmation runs.

Figure 10 shows the box plots of the four responses vs the control parameters, categorized in Ranks 1 and 2 for each response. *R*_a_ against ET and EW is shown in Figure 10a, *R*_q_ against ET and EW is shown in Figure 10b, A2N_95_ against EW and ST is shown in Figure 10c, and *P*_CT_ against EW and RO is shown in Figure 10d. In the majority of cases, the values seem to be scattered, while they are concentrated within the same range only in specific cases. For *P*_CT_ values, they are scattered over a wider amplitude.

**Figure 10:**
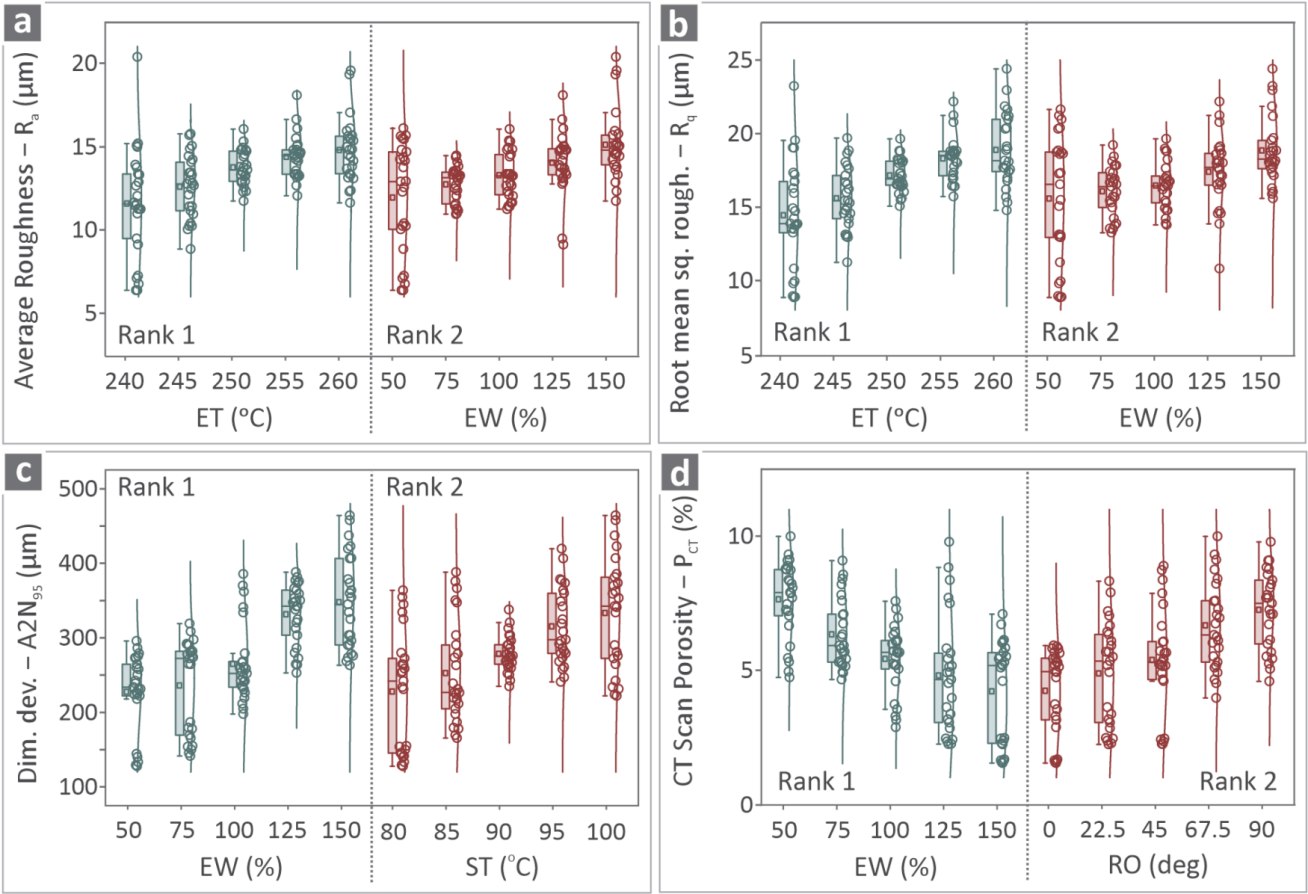
Box plots of (a) *R*_a_ vs ET and EW, (b) *R*_q_ vs ET and EW, (c) A2N_95_ vs EW and ST, and (d) *P*_CT_ vs EW and RO.

Figure 11a–e shows the 3D surface plots of the response metrics vs the two most important control parameters for each metric, i.e., *R*_a_ vs ET and EW (Figure 11a), *R*_q_ vs ET and EW (Figure 11b), A2N_95_ vs RO and DV (Figure 11c), A2N_95_ vs EW and ST (Figure 11d), and *P*_CT_ vs RO and EW (Figure 11e). In Figure 11f, the bars show the correlation factor versus the four quality indicators: *R*_a_, *R*_q_, A2N_95_, and *P*_CT_. For all responses, negative correlation factor values were found compared to the tensile strength, indicating an inverse relationship between them; i.e., as one parameter increases, the other decreases. This is a critical finding derived from the analysis, documenting that the increase in the values of the response metrics (higher roughness, porosity, and geometrical deviation) leads to inferior tensile strength in the samples.

**Figure 11:**
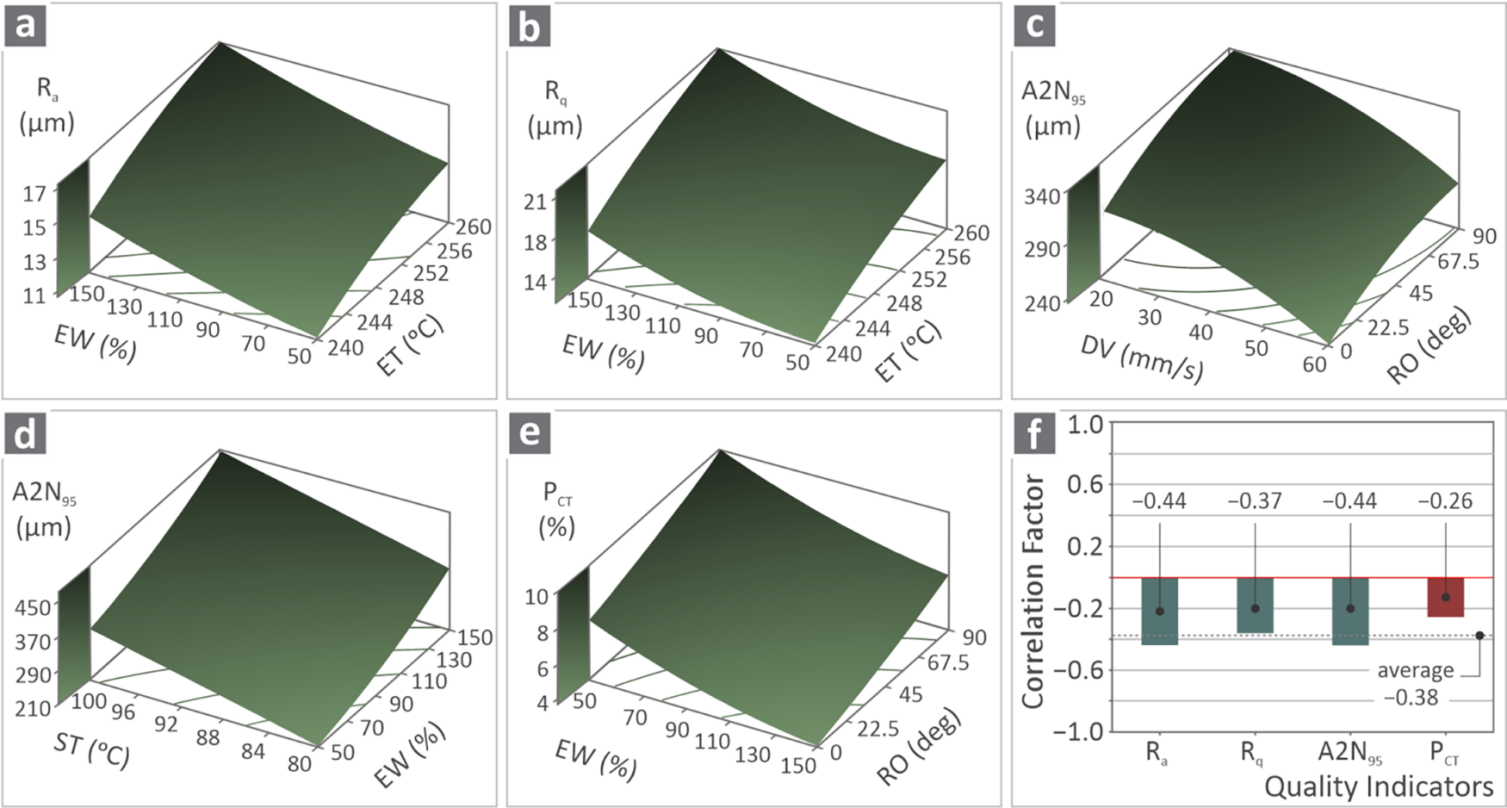
Surface plots of (a) *R*_a_ vs ET and EW, (b) *R*_q_ vs ET and EW, (c) A2N_95_ vs RO and DV, (d) A2N_95_ vs EW and ST, and (e) *P*_CT_ vs RO and EW, and (f) graph of correlation factor vs quality indicators.

## 4 Discussion

Optimizing porosity, dimensional accuracy, and surface roughness in 3D-printed ASA components fabricated through MEX can be advantageous for several applications, particularly in outdoor or automotive contexts where ASA’s weather-resistant properties are crucial. Efforts to enhance the porosity and surface roughness have resulted in components with superior performance. Minimizing porosity reduces the internal void space that can serve as initiators of fractures or mechanical failures, thereby enhancing fatigue resistance and structural integrity. Improving geometrical accuracy improves the functionality of the parts. Similarly, reducing surface roughness diminishes crack initiation by lowering stress concentrations on the surface, thus improving durability. From a weathering perspective, decreased porosity mitigates moisture absorption in ASA, potentially reducing the risk of corrosion, which is significant for outdoor applications. Furthermore, reduced surface roughness enhances aesthetics and provides a more uniform base for coatings or painting, resulting in more effective sealing and waterproofing properties in automotive and outdoor applications. A reduced surface area decreases frictional forces, potentially extending the service life and reducing wear and tear. Additionally, a smoother surface is less susceptible to biological contaminants, such as fungal growth, which can degrade the surface integrity and cause contamination, a critical consideration for outdoor applications.

The selection of a Taguchi design over alternative methodologies such as response surface methodology (RSM) or central composite design (CCD) for optimizing the ASA MEX process parameters was deemed a robust parameter design solution. While RSM and CCD are typically favored for modeling complex, nonlinear response surfaces with specific quadratic and interaction terms are most beneficial. After the significant factors have been substantially narrowed down, it was imperative to employ a Taguchi-type design in this instance. This approach was necessary, as six parameters were evaluated concurrently to establish an initial design framework, which could subsequently be refined to identify a reduced set of critical parameters for further optimization using more sophisticated tools in future research. The Taguchi method, with its application of orthogonal arrays, provides a practical means of examining main effects with statistical confidence by minimizing the number of experimental runs required to determine the effects of variables. Consequently, this research utilized the Taguchi design as an effective and rigorous screening method to identify the factors exerting the most significant impact on quality measures.

Still, the limitations of the L25 array primarily allow a reliable estimation of main effects. Quadratic terms cannot be independently identified from the design and are therefore reported only as indicative regression trends based on replication. To clarify the role of interactions, pairwise interaction terms were explicitly tested during regression analysis. None reached statistical significance, and given the aliasing restrictions of the L25 design, they were excluded from the final models. To increase transparency, interaction plots are now provided in the Supporting Information for all responses (*R*_a_, *R*_q_, A2N95, *P*_CT_), which illustrate the absence of strong factor interactions. The factors identified as important during the ANOVA of the Taguchi results could then be further investigated through a more comprehensive sequential RSM study aimed at explicitly modeling quadratic effects and factor interactions, thereby achieving full optimization with resources in any subsequent study.

The rationale for employing regression analysis in this research context is to transition from empirical observation to predictive knowledge, aligning with the established academic principles governing both classification modes. The primary justification for utilizing regression analysis following a Taguchi-designed experiment is to develop empirical mathematical models that quantitatively describe the complex and nonlinear relationships among the six critical MEX process parameters and the quality responses of the ASA parts, specifically porosity, surface roughness, and dimensional accuracy. The Taguchi methods facilitate the efficient identification of the relative importance of factors and propose a parameter combination that optimizes the mean performance. However, Taguchi methods rely solely on data and do not yield a continuous predictive function. Regression analysis addresses this limitation by providing a transfer function that predicts all response values for any given combination of input parameters within the study’s range. This modeling also enhances the understanding of dynamic processes influencing the quality responses of the ASA parts, capturing not only the main effects but also two-factor interactions and quadratic effects, which are essential for understanding the complex thermal and rheological behavior of ASA during the extrusion and deposition process. Additionally, statistical significance tests are conducted for all terms in the regression equation, enabling researchers to optimize further by eliminating insignificant variables, thereby refining the regression model. Ultimately, the regression model facilitates powerful multiobjective optimization to identify a process window that achieves the optimal balance between the conflicting objectives of minimizing porosity, improving surface finish, and enhancing dimensional accuracy, advancing ASA parts toward functional or engineering applications.

The need for the minimization of necessary experimental effort (number of tests), data processing, and specimen fabrication led to the formation of the Taguchi L25 DOE while still securing the derivation of reliable results. One hundred and twenty-five specimens were examined for each of the 25 experimental runs, corresponding to five replicates. In addition, two experimental runs were conducted, creating an additional ten tested specimens; however, the number of samples tested was significantly less than they would have been in the case of a full factorial design.

Based on the derived experimental data, the parameters with the greatest impact on the responses were ET for *R*_a_ and *R*_q_ and EW for A2N_95_ and *P*_CT_. On the other hand, DV had the least impact on *R*_a_ and *R*_q_, ET had the least impact on A2N_95_, and ST had the least impact on *P*_CT_. The tensile strength derived from previous research and obtained for correlation purposes seemed to be most affected by RO and least influenced by LH.

It should be mentioned that Run 23, with 150% EW, 45.0° RO, 20 mm/s DV, 0.15 mm LH, 260 °C ET, and 95 °C ST as printing parameters, presented the highest *R*_a_ (17.37 μm) and *R*_q_ (21.71 μm). The highest A2N_95_ levels were revealed by Run 24 (437.76 μm), with 150% EW, 67.5° RO, 30 mm/s DV, 0.20 mm LH, 240 °C ET, and 100 °C ST as printing parameters. The highest *P*_CT_ levels were detected in Run 4 (9.08%), with 50% EW, 67.5° RO, 50 mm/s DV, 0.25 mm LH, 255 °C ET, and 95 °C ST as printing parameters.

The lowest *R*_a_, *R*_q_, and A2N_95_ levels (6.79, 9.29, and 134.95 μm, respectively) were revealed in the case of Run 1, with 50% EW, 0.0° RO, 20 mm/s DV, 0.10 mm LH, 240 °C ET, and 80 °C ST as printing parameters; the optimum 3D printing settings achieved a recution in the *R*_a_ surface roughness by 256% (reduced from 17.37 μm in Run 23 to 6.79 μm in Run 1) and 234% for the *R*_q_ metric (reduced from 21.71 μm in Run 23 to 9.29 μm in Run 1). The A2N_95_ was also reduced by an impressive 324% (from 437.76 μm in Run 24 to 134.95 μm in Run 1), leading to much more geometrically accurate 3D-printed ASA parts. As far as *P*_CT_ is concerned, the lowest percentage was detected in the case of Run 21 (1.61%), with printing parameters of 150% EW, 0.0° RO, 50 mm/s DV, 0.30 mm LH, 250 °C ET, and 85 °C ST. The reduction in the porosity compared to Run 4 (9.08%) was 564%. Such findings highlight the importance of selecting a proper set of 3D printing settings, as they significantly impact the quality of the manufactured components. Better surface roughness and geometrical accuracy were achieved with lower values on 3D printing settings, while reduced porosity was achieved with high values of some parameters (EW, LH) and median values in others (DV, ET, ST).

The MEP in Figure 8, apart from the trends for the responses examined herein, also presents a correlation with the tensile strength. For the surface roughness metrics, a clear trend is observed, with an increase in EW and RO, leading to higher *R*_a_ and *R*_q_ values, and a reduction in the tensile strength of the parts. The increase in ET increases the surface roughness metrics, but it does not seem to have a clear effect on the tensile strength, probably because this control parameter is not ranked as an important one for the strength metric. Regarding ST, its increase leads to increased surface roughness and strength at the same time. Still, it is ranked low, therefore not highly affecting the overall effect. For dimensional deviation and porosity, their increase with increased RO values leads to a decrease in tensile strength. The increase in EW leads to increased geometrical deviation and reduced porosity while also decreasings the strength. The remaining 3D printing settings have a mild effect on tensile strength.

The box plots presented in Figure 10 show data distributed with whiskers and, in most cases, centered around specific values. Only in a few cases are data points outside the whiskers, thus indicating outliers (data points that significantly differ from the data set). By observing the box plots, the variability in the control parameter values can be observed.

The effects of 3D printing parameters on the quality metrics and their correlation with the tensile strength are more clearly presented in Figure 11f, which shows the correlation factor between the quality metrics and strength. All metrics have negative correlation factors, showing that as their values increase (leading to reduced parts’ quality), the tensile strength decreases. Lower (negative) values were found for *R*_a_ and A2N_95_, followed by *R*_q_, and finally, *P*_CT_ had the highest value (although still a negative value, as mentioned). These findings were more or less expected after the analysis of the MEP; however, in these graphs, they are clearer and more quantified. This is a quantified solid finding, relating the quality of the 3D-printed ASA samples to their strength, with the analysis showing, as mentioned, that inferior quality, in terms of surface roughness, dimensional accuracy, and porosity, leads to parts with lower tensile strength.

The selection of the RQRM regression model over the LRM and QRM was made after evaluating the respective ANOVA metrics. LRM did not manage to achieve sufficient predictions, while the difference in the prediction performance between the RQRM and QRM was negligible. The *R* values of RQRM and QRM differed by less than 2%, remaining within the limit of statistical error. Therefore, reliable predictions can be made in this experimental scenario with the formulas produced by RQRM, without the need for the more advanced and complex QRM formulas. Overall, the RQRM model was chosen over the QRM model due to its simplicity. The aim was to find a simpler regression that would efficiently model the specific experimental scenario. In this direction, as RQRM was proven efficient, there was no valid reason to elaborate the research with more complex and sophisticated modeling tools that would require additional calculation effort and power to be resolved.^140^ The results showed that the performance of RQRM and QRM differed by less than 2%, which is well within the statistical error margin, while both models clearly outperformed LRM. Therefore, RQRM was selected because it provided almost the same predictive accuracy as the QRM, while being simpler and easier to use. The additional terms of the QRM did not bring any significant improvement, so the reduced model was considered the more practical choice.

The *R* values for the surface roughness response metrics were in the range of 80%, while those for A2N_95_ and *P*_CT_ were in the range of 90% (a maximum *R* value of 92%). These values characterize the prediction method as trustworthy. On the other hand, the LRM model yielded lower *R* values, ranging between 73% and 86%, which could be considered acceptable results, but still not as impressive as those of the other two models. As far as prediction accuracy is concerned, as presented in the validation table of the confirmation runs, the error remained between 5 and 10% in all of the cases, which is a very acceptable result, verifying the effectiveness of the prediction models produced by the analysis.

Despite these excellent results, it should be noted that for specific response metrics and 3D printing settings (control parameters), as indicated by the ANOVA tables, low *F-*values were found in combination with *P* values greater than 0.05. This is the case for both the EW and ST 3D printing settings (control paramaters), as well as for surface roughness metrics. The same applies, for the control paramaters EW, DV, and ET regarding the A2N_95_ response, and DV, ET, and ST for the *P*_CT_ response. A low *F*-value means that the group means are not significantly different compared to the variability within each group, while a high *p*-value (*p* > 0.05) suggests no statistically significant difference among the groups. Still, the overall *F*-values for all four responses are high (Table 8), and therefore, the findings can be considered solid, taking into account the remaining findings of the analysis mentioned above.

## 5 Conclusions

This research aimed to optimize parameters for 3D-printed samples of the ASA material, focusing on their quality metrics. For this purpose, the Taguchi L25 DOE was utilized, with EW (%), RO (deg), LH (mm), DV (mm/s), ET (°C), and ST (°C) 3D printing parameters as the input parameters (control parameters) and *R*_a_, *R*_q_, A2N_95_, and *P*_CT_ as the quality metrics to be optimized. The attempt at parameter optimization and examination of their influence on the chosen responses proved promising for future applications and effective for current ones. Valuable results were obtained, and valid assumptions were made regarding 3D printing of the ASA material and how to optimize it. The adjustment of the 3D printing parameters provided noticeable diversification of the four responses and created potential for further research to expand the existing literature. The main findings are summarized as follows:

- Out of the 25 experimental runs, Run 1 (50% EW, 0° RO, 0.1 mm LH, 20 mm/s DV, 240 °C ET, and 80 °C ST) had the lowest surface roughness and A2N_95_ levels (*R*_a_ 256%, *R*_q_ 234%, and A2N_95_ 324% difference compared to the highest values found in Run 23 for surface roughness and Run 24 for A2N_95_).
- Run 21 (150% EW, 0° RO, 0.3 mm LH, 50 mm/s DV, 250 °C ET, and 85 °C ST) had the lowest *P*_CT_ levels (564% difference compared to the highest values found in Run 4).
- The parameter that most affected the *R*_a_ and *R*_q_ responses was found to be ET, which was ranked first, while DV was the least influential.
- The greatest impact of A2N_95_ and *P*_CT_ was caused by EW, while ET and ST ranked sixth.
- The suitability of RQRM was also a positive outcome, as the utilization of a more complex model, such as QRM, was proven necessary in the specific experimental scenario.
- The prediction models were more sufficient, with *R* values of around 80%, making them reliable for employement in real-life fields.
- This was also confirmed by the two additional confirmation runs, in which the deviation between the predicted and actual values was less than 10%.
- The correlation between the quality metrics and tensile strength showed a clear impact, presented in both the MEP and the correlation factor graph. The worsening of all four quality metrics examined (an increase in their values) affects the tensile strength in a negative way.

Additional work could be conducted in the future using different printing parameters and levels and different experimental designs. Broadening the parameter range increases the merit, making the findings applicable to a larger range of values and, by extension, to various applications. The modeling provided herein is expected to operate within the calculated accuracy when parameter values are selected within the range examined in the current research. Therefore, there is a strong potential in studying a wider range of values. The quality metrics, as documented in the research, affect the mechanical performance of the 3D-printed samples. Therefore, improving the quality should also be favorable for the mechanical performance. Studying the mechanical performance with the same control parameters and levels and correlating the results can be addressed in future work. This would further document and expand the benefits of the current findings for real-life applications, as well.

## Supplementary Material



## Data Availability

The raw/processed data required to reproduce these findings cannot be shared because of technical or time limitations.

## Notes

Measured *R*_a_, *R*_q_, A2N_95_, and *P*_CT_ for each experimental run and five replicas per run (Table S1); Measured *R*_a_, *R*_q_, A2N_95_, and *P*_CT_ for the five replicas of the confirmation experimental runs (Table S2); roughness interaction plots (*R*_a_ and *R*_z_) (Figure S1); and dimensional deviation and porosity interaction plots (Figure S2) (PDF)
