# Supplementary material for: Optimization of Roughness, Dimensional Conformity, and Porosity of 3D-Printed ASA with MEX: Impact of Critical Process Control Parameters
Source: ACS Omega. 2025 Nov 18;10(47):57286–305. doi: 10.1021/acsomega.5c07437 (PMC12676300; doi:10.1021/acsomega.5c07437)
Supplement: Supplementary file 1 [file ao5c07437_si_001.pdf]

Optimization of roughness, dimensional conformity, and porosity of 3D printed ASA with MEX: impact of critical process control parameters

Dimitrios Sagris [a], Constantine David [a], Markos Petousis [b], Nektarios K. Nasikas [c], Nikolaos Mountakis [b], Maria Spyridaki [b], Nectarios Vidakis [b] *

[a] Dept. of Mechanical Engineering, International Hellenic University, Serres Campus, 62124, Greece

[b] Dept. of Mechanical Engineering, Hellenic Mediterranean University, Heraklion 71410, Greece

[c] Division of Mathematics and Engineering Sciences, Department of Military Sciences, Hellenic Army Academy, 16673 Vari, Attica, Greece

* Corresponding author: Nectarios Vidakis, e-mail: vidakis@hmu.gr, Tel.: +302810379227

S.1. Micro-computed tomography methodology

The porosity values were calculated using the VGDefX algorithm in void analysis mode, a widely used approach for CT-based porosity quantification. This method identifies voids based on grayscale intensity differences in the CT scan data and segments them using a probability threshold of 1.00, ensuring the detection of all distinguishable pores. The threshold value was selected based on prior studies using similar segmentation techniques for polymer-based composites and was chosen to maximize sensitivity while avoiding false detections. The total porosity was computed as the ratio of the detected void volume to the total volume of the specimen, including both the solid material and the voids. This approach ensures a quantitative assessment of porosity, as it accounts for the entire specimen volume rather than isolating the voids from the bulk material. To maintain accuracy, noise reduction was set to 'Low' to preserve fine details, and surface sealing was disabled to prevent artificial modifications to the porosity data.

The porosity analysis was based on the assumption that all detected voids represent actual material defects rather than imaging artifacts. A minimum detectable pore size of 0.10 μm was set, corresponding to the CT scan's resolution limit, ensuring that only pores above this threshold were quantified. The analysis area was defined as 'Internal cleaning, small', meaning that only internal voids were considered, while surface irregularities were excluded to avoid overestimating porosity due to surface roughness or edge effects. Additionally, CT scan artifacts were not removed, as the objective was to capture the full porosity distribution, acknowledging that minor imaging artifacts could be present but do not significantly impact the overall results.

Regarding the limitations of the imaging technique, the CT scan was performed at 120 kV and 700 μA (source power of 84 W), with 1600 rotational measurement positions, three multiple projections per position, and an exposure time of 66 milliseconds per position, ensuring high scanning precision and reproducibility while minimizing potential imaging artifacts. The voxel size was 0.0159 mm, meaning that pores smaller than this resolution might not be accurately detected. The absence of beam hardening correction could introduce minor contrast variations, particularly near the specimen's edges. Additionally, as CT imaging relies on grayscale intensity differences, very fine porosity may be underestimated if the contrast between the polymer matrix and voids is insufficient. The CT scan parameters were selected based on optimal settings established in previous studies and standardized measurement protocols, ensuring reliable porosity quantification and comparability with past research on similar materials. By maintaining consistent measurement parameters, this approach enables direct comparisons across different studies while ensuring high contrast, resolution, and minimal imaging artifacts.

S.2. Taguchi DOE

The Taguchi method is first used to select cases for modeling from the orthogonal array. Classical experimental design methods are too complex and thus not easy to use. In addition, a large number of experiments or simulations must be carried out when the number of process parameters increases.

The Taguchi method uses a special design of orthogonal arrays to study the entire parameter space so that far fewer experiments are required to solve the problem [1]. The total degree of freedom (DOF) is normally used to select an orthogonal array. The DOF for each factor is the number of factor levels minus 1 [2]; This value must be lower than the DOF of the selected orthogonal array.

The Taguchi method is later also used to analyze how different modeled parameters influence the solution to the problem and to obtain the best combination of parameters [3]. It has been applied in many research fields [4] mainly because of its proven success [5–9]. The method provides the order of importance of independent parameters for dependent objective functions [10]. It also defines a set of parameters that will produce the best-case and worst-case scenarios.

The calculation procedure of the Taguchi method consists of the calculation of signal-to-noise (S/N) ratios for each investigated factor, the calculation of delta values from S/N ratios, and the determination of the order of each factor.

The S/N ratio is defined in three different forms: the larger is the better, the lower is the better, and the nominal is the best [11]. These equations are listed below:

The larger the better [12]:

$$S/_N = -10\log\left(\frac{1}{n}\sum_{i=1}^{n}\frac{1}{Y_i^2}\right) \quad (1)$$

Smaller the better [5]:

$$S/_N = -10\log\left(\frac{1}{n}\sum_{i=1}^{n}Y_i^2\right) \quad (2)$$

Nominal the best [13,14]:

$$S/_N = 10\log\left(\frac{\mu^2}{\sigma^2}\right) \quad (3)$$

Where μ represents the means and σ represents the standard deviation, and Yi represents the resulting value for the i_{th} objective function.

The delta values are calculated as the difference between the maximum and minimum S/N values of each parameter and then ranked. The largest delta value represents the most effective parameter, and the rank is obtained from this order.

S.3. ANOVA Statistical Technique

ANOVA is a widely known and used statistical technique that allows the interpretation of experimental results by obtaining the contribution ratio of each parameter. ANOVA was used to examine the significance of each parameter for the problem to be solved using computational fluid dynamics (CFD). The calculation steps used in the ANOVA were as follows.

The total sum of squares (SS_T) was calculated by Ref. [15]:

$$SS_T = \sum_{i=1}^{N}(Y_i - \overline{Y})^2 \quad (4)$$

where N is the number of cases in the orthogonal array, Y_i is the experimental and numerical results for the i_{th} experiment, and

$$\overline{Y} = \frac{1}{N}\sum_{i=1}^{N}Y_i \quad (5)$$

The total sum of the squared deviations SS_T consists of the sum of the squared error SS_e and the sum of the squared deviations SS_P due to each process parameter; therefore, SS_P was defined as [15]

$$SS_P = \sum_{j=1}^{t} \frac{\left(SY_j\right)^2}{t} - \frac{1}{N}\left[\sum_{i=1}^{N} Y_i\right]^2 \tag{6}$$

where P is one of the parameters, j is the level number of parameter P, t is the repetition of each level of parameter P, and SY_j is the sum of the experimental results involving parameter P and level j. The sum of squares from the error parameter SS_e is [15]

$$SS_e = SS_T - SS_A - SS_B - SS_C - SS_D - SS_E \tag{7}$$

The total degree of freedom was $D_T = N - 1$, and the degree of freedom of each tested parameter was $D_P = N - 1$. The variance of the parameters tested was $V_P = SS_P/D_P$. The F-value for each design parameter is simply the ratio of the mean of the squares deviations to the mean of the squared error $F_P = V_P/V_e$. The percentage contribution ρ, was calculated as follows[15]:

$$\rho_P = \frac{SS_P}{SS_T} \tag{8}$$

S.4. Regression Model

Three possible regression models can be utilized, and are presented below in Equations 9, 10, and 11. However, in this study, a reduced quadratic regression model was employed.

The Linear Regression Model (LRM) for each response is calculated:

$$Y_k = a_k + \sum_{i=1}^{n} b_{i,k} x_i + e_k \tag{9}$$

The Reduced Quadratic Regression Model (RQRM) for each response is calculated:

$$Y_k = a_k + \sum_{i=1}^{n} b_{i,k} x_i + \sum_{i=1}^{n} c_{i,k} x_i^2 + e_k \tag{10}$$

The Quadratic Regression Model (QRM) for each response is calculated:

$$Y_k = a_{i,k} + \sum_{i=1}^{n} b_{i,k} x_i + \sum_{i=1}^{n} c_{i,k} x_i^2 + \sum_{i} \sum_{j} d_{ij,k} x_i x_j + e_k \tag{11}$$

Were, k represents the response output (i.e., Average roughness R_a, Root mean square roughness R_q, Actual-to-Nominal dimensional deviation at 95% $A2N_{95}$, Porosity CT Scan P_{CT}), a the constant value, b the coefficients of the linear terms, c the coefficients of the square terms, d the coefficients of the two way interaction terms, e the error and x_i the six (n=6) control

parameters (i.e. Extrusion Width EW, Raster Orientation RO, Layer Height LH, Deposition Velocity DV, Extruder Temperature ET, Substrate Temperature ST).

S.5. Experimental Results

Table S1. Measured R_a, R_q, $A2N_{95}$, and P_{CT} for each experimental run and five replicas per run

A/A	Run	Ra (μm)	Rq (μm)	A2N95 (μm)	PCT (%)
1	1	7.27	9.95	127.68	4.74
2		6.39	8.86	129.27	4.95
3		6.39	8.94	133.37	5.32
4		7.12	9.82	140.11	5.45
5		6.78	8.88	144.30	5.89
6	2	10.41	13.10	217.89	6.66
7		10.26	13.13	224.66	7.04
8		8.87	11.24	226.90	7.20
9		10.25	12.94	231.16	7.90
10		10.04	12.99	238.75	8.32
11	3	12.57	15.49	250.53	7.47
12		12.29	15.06	259.92	7.87
13		13.76	17.32	264.77	8.37
14		12.93	17.27	273.10	8.74
15		12.90	16.55	275.67	8.89
16	4	14.37	18.61	257.86	8.23
17		14.81	18.85	273.02	8.76
18		14.16	18.75	279.26	9.12
19		14.25	18.74	286.25	9.32
20		14.68	18.64	295.84	9.99
21	5	15.43	20.40	222.17	7.25
22		16.10	21.64	223.91	7.70
23		15.51	20.31	231.48	8.08
24		15.68	20.97	233.73	8.78
25		15.64	21.22	242.21	9.10
26	6	12.06	15.73	264.79	5.19
27		13.15	17.34	272.28	5.29
28		14.47	19.22	275.82	5.67
29		12.68	18.44	280.90	5.81

30		13.55	18.10	290.07	5.93
31	7	13.31	17.00	141.46	4.93
32		14.39	17.82	145.23	5.35
33		12.37	15.31	149.97	5.69
34		13.39	17.42	154.05	5.93
35		13.82	15.68	155.11	6.34
36	8	11.28	13.50	165.72	4.66
37		11.58	14.23	169.43	4.90
38		10.99	13.27	178.04	5.15
39		11.27	13.75	179.43	5.31
40		11.47	13.54	186.96	5.42
41	9	10.96	13.88	265.04	6.25
42		14.07	17.84	273.84	6.74
43		12.49	15.44	278.13	7.07
44		12.98	16.00	280.71	7.60
45		11.16	14.98	290.84	8.41
46	10	13.47	16.70	281.93	7.10
47		13.20	16.40	291.95	7.81
48		13.52	16.91	297.62	8.21
49		13.27	16.68	308.36	8.57
50		13.32	16.55	319.25	9.09
51	11	12.81	16.55	241.03	2.88
52		12.12	14.78	246.96	3.15
53		11.42	14.22	260.90	3.29
54		12.67	15.30	268.18	3.55
55		11.43	15.43	279.07	3.73
56	12	15.32	18.79	334.08	5.19
57		14.41	17.13	340.96	5.50
58		16.05	19.65	361.94	5.69
59		14.93	18.83	369.92	6.09
60		14.53	18.25	385.57	6.44
61	13	13.37	16.97	230.99	5.20
62		13.34	16.90	233.94	5.64
63		13.23	16.40	242.03	5.72
64		13.35	16.44	254.80	6.05

65		13.26	16.40	258.77	6.65
66		11.64	14.79	197.67	6.11
67		15.36	16.80	204.91	6.32
68	14	14.80	18.98	211.38	6.96
69		12.31	17.65	219.19	7.29
70		14.84	16.19	226.31	7.58
71		13.37	16.74	235.06	4.59
72		11.26	13.79	242.54	5.05
73	15	12.04	14.93	252.13	5.25
74		12.65	15.96	259.78	5.67
75		11.66	13.88	266.86	5.98
76		13.14	17.56	286.25	4.93
77		13.10	17.70	303.64	5.13
78	16	14.51	19.14	312.68	5.43
79		14.16	18.86	320.62	5.64
80		13.69	18.08	337.86	5.82
81		14.98	19.02	324.85	2.84
82		9.14	10.81	341.93	3.06
83	17	13.75	17.20	348.81	3.17
84		12.88	13.87	364.15	3.37
85		9.49	14.73	377.22	3.63
86		14.88	18.66	342.28	2.25
87		12.77	14.57	353.54	2.26
88	18	13.46	16.70	359.24	2.35
89		13.42	16.24	373.52	2.42
90		13.31	14.57	381.35	2.46
91		13.90	16.51	253.07	3.97
92		14.83	17.98	263.95	4.52
93	19	14.81	18.18	264.59	4.66
94		14.79	18.02	272.51	4.91
95		14.73	16.53	279.95	5.17
96		18.09	22.17	347.51	7.50
97	20	16.08	18.59	350.06	7.70
98		15.04	17.13	366.35	8.36
99		15.51	20.77	375.71	8.83

100		16.63	21.25	388.22	9.79
101		11.74	15.60	263.11	1.54
102		13.80	18.27	273.35	1.57
103	21	12.33	16.03	278.59	1.62
104		12.86	18.13	290.30	1.64
105		13.62	15.90	303.38	1.68
106		14.54	18.84	268.47	2.24
107		14.58	18.80	275.82	2.28
108	22	15.11	17.76	290.14	2.38
109		14.66	18.01	296.28	2.46
110		14.79	18.52	303.44	2.48
111		14.99	18.16	359.85	4.60
112		19.56	24.40	373.54	5.18
113	23	17.04	21.18	378.50	5.23
114		19.32	21.85	406.34	5.66
115		15.94	22.95	419.57	6.07
116		20.37	23.23	406.75	5.10
117		13.32	16.29	422.70	5.31
118	24	13.91	17.34	437.33	5.52
119		15.19	19.05	457.88	5.84
120		15.08	19.55	464.15	6.02
121		15.78	19.71	325.12	5.43
122		14.22	17.16	334.48	5.51
123	25	14.44	17.61	345.07	6.13
124		15.70	18.05	354.52	6.71
125		15.05	18.85	363.66	7.10
	Min:	6.39	8.86	127.68	1.54
	Max:	20.37	24.40	464.15	9.99
	Average:	13.426	16.873	281.503	5.685

Table S2. Measured R_a, R_q, $A2N_{95}$, and P_{CT} for the five replicas of the Confirmation experimental runs

A/A	Run	Ra (μm)	Rq (μm)	A2N95 (μm)	PCT (%)
1	26	12.53	15.19	250.35	5.41
2		11.76	15.95	245.79	5.51

3		11.97	14.67	234.65	5.63
4		12.85	15.68	236.87	5.69
5		12.39	14.97	253.48	5.25
6		15.10	18.77	317.45	7.52
7		15.81	19.93	341.73	7.36
8	27	15.53	19.04	329.16	6.90
9		16.55	19.37	332.76	7.01
10		16.01	20.09	318.05	7.26
	Min:	11.76	14.67	234.65	5.25
	Max:	16.55	20.09	341.73	7.52
	Average:	14.050	17.366	286.029	6.354

S.6. ANOVA

Figure S1 and Figure S2 present the interaction plots for the surface roughness Ra and the dimensional accuracy. As shown, and especially in specific ranges of the control factor values, little or no interaction can be observed, as lines seem to follow a rather parallel or non-crossing pattern.

This means that the effect of one factor on the response variable does not depend heavily on the level of another factor. These interactions between the control factor levels should be considered when selecting the levels of the 3D printing settings.

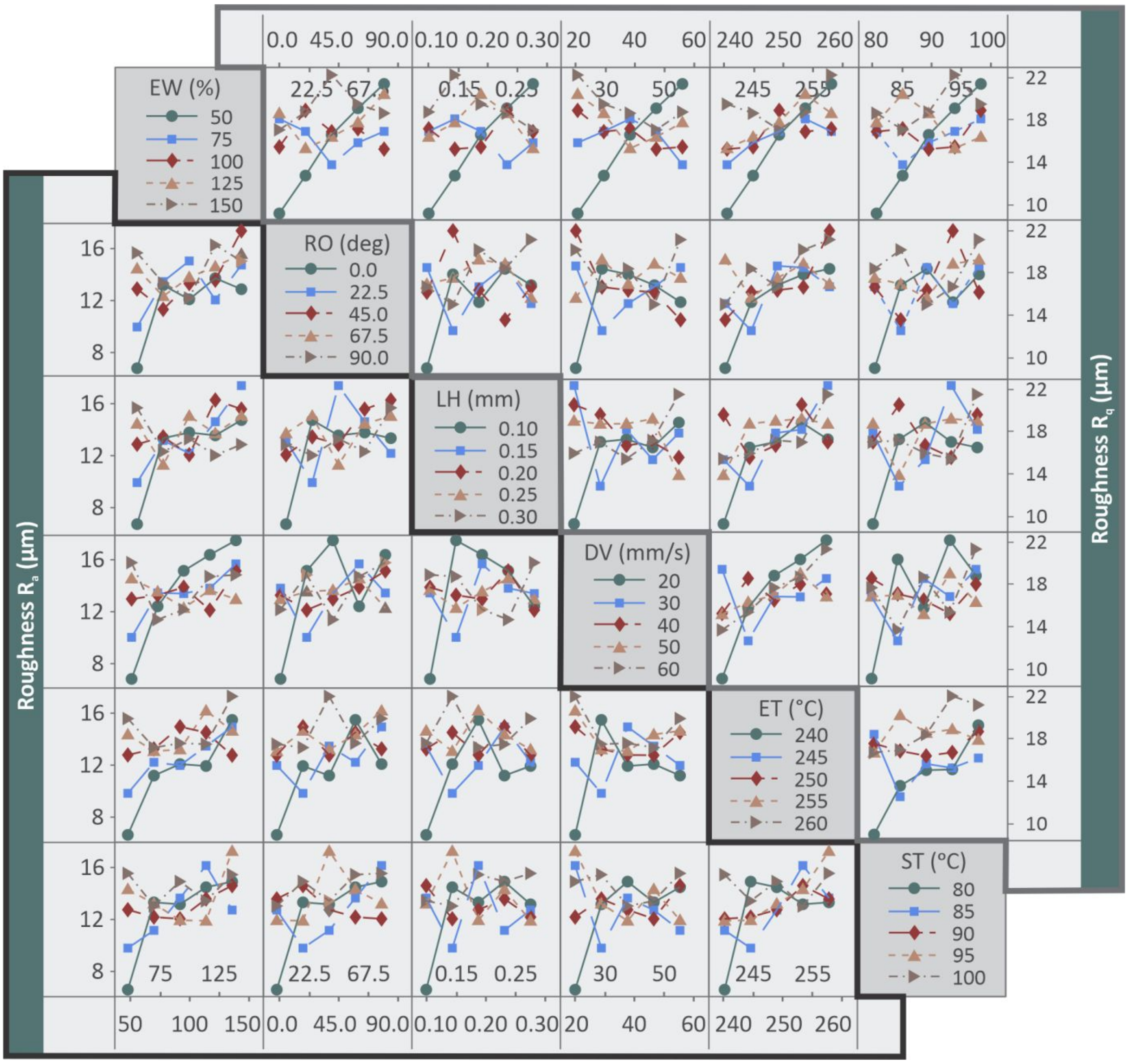

Figure S1. Roughness interaction plots (Ra and Rz).

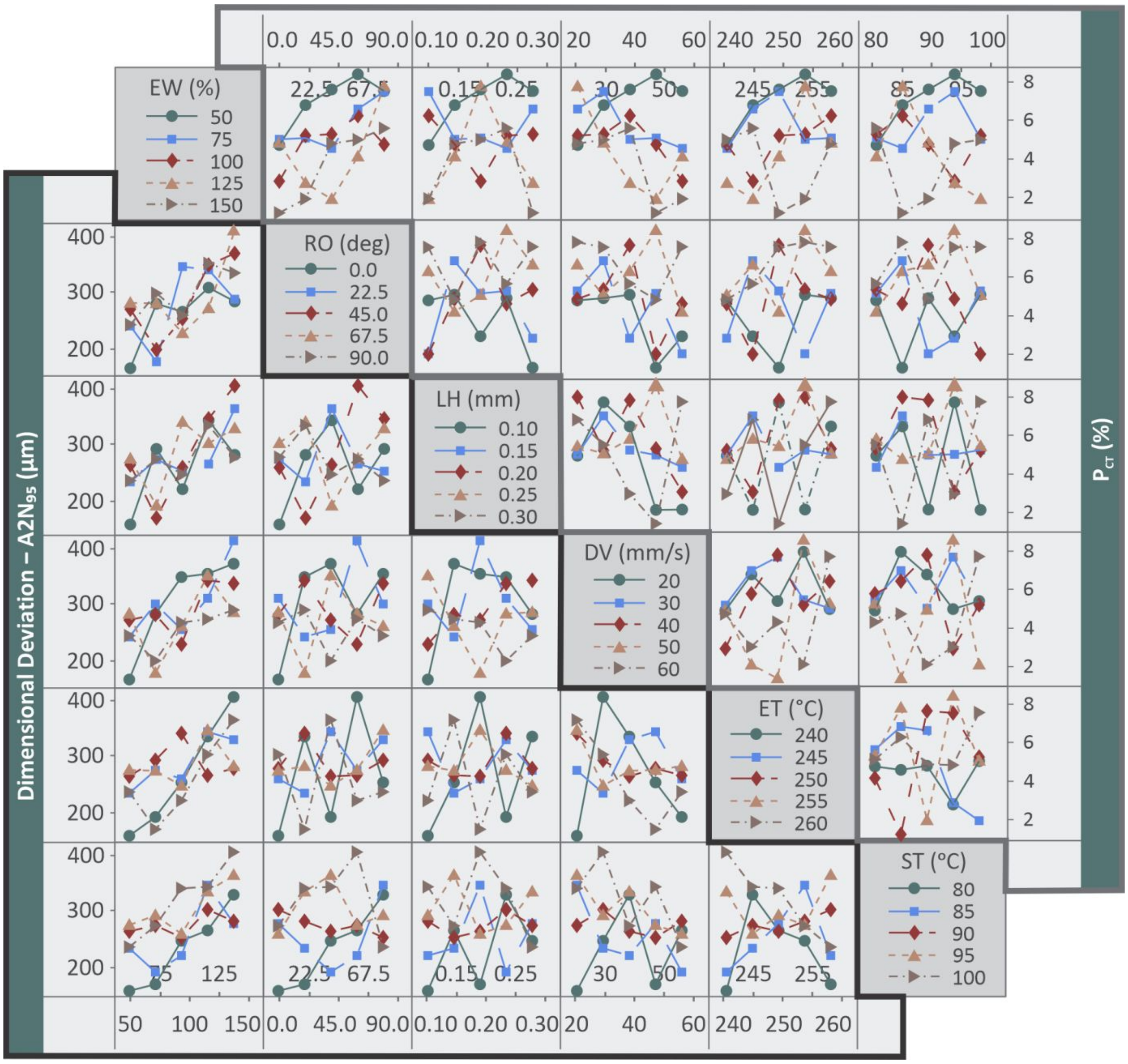

Figure S2. Dimensional deviation and porosity interaction plots.

References

(1) Yang, W. H.; Tarng, Y. S. Design Optimization of Cutting Parameters for Turning Operations Based on the Taguchi Method. *J Mater Process Technol* **1998**, *84* (1–3), 122–129. https://doi.org/10.1016/S0924-0136(98)00079-X.

(2) Ning, M.; Mengjie, S.; Mingyin, C.; Dongmei, P.; Shiming, D. Computational Fluid Dynamics (CFD) Modelling of Air Flow Field, Mean Age of Air and CO2 Distributions inside a Bedroom with Different Heights of Conditioned Air Supply Outlet. *Appl Energy* **2016**, *164*, 906–915. https://doi.org/10.1016/j.apenergy.2015.10.096.

(3) Niu, B.; Shi, M.; Zhang, Z.; Li, Y.; Cao, Y.; Pan, S. Multi-Objective Optimization of Supply Air Jet Enhancing Airflow Uniformity in Data Center with Taguchi-Based Grey Relational Analysis. *Build Environ* **2022**, *208*, 108606. https://doi.org/10.1016/j.buildenv.2021.108606.

(4) Taguchi, G. Introduction to Quality Engineering, Asian Productivity. *Organization* **1990**, 1–24.

(5) Arslanoglu, N.; Yigit, A. Experimental Investigation of Radiation Effect on Human Thermal Comfort by Taguchi Method. *Appl Therm Eng* **2016**, *92*, 18–23. https://doi.org/10.1016/j.applthermaleng.2015.09.070.

(6) Chang, C.-W.; Kuo, C.-P. Evaluation of Surface Roughness in Laser-Assisted Machining of Aluminum Oxide Ceramics with Taguchi Method. *Int J Mach Tools Manuf* **2007**, *47* (1), 141–147. https://doi.org/10.1016/j.ijmachtools.2006.02.009.

(7) Pinar, A. M.; Uluer, O.; Kırmaci, V. Optimization of Counter Flow Ranque–Hilsch Vortex Tube Performance Using Taguchi Method. *International Journal of Refrigeration* **2009**, *32* (6), 1487–1494. https://doi.org/10.1016/j.ijrefrig.2009.02.018.

(8) Özel, S.; Vural, E.; Binici, M. Optimization of the Effect of Thermal Barrier Coating (TBC) on Diesel Engine Performance by Taguchi Method. *Fuel* **2020**, *263*, 116537. https://doi.org/10.1016/j.fuel.2019.116537.

(9) Tutar, M.; Aydin, H.; Yuce, C.; Yavuz, N.; Bayram, A. The Optimisation of Process Parameters for Friction Stir Spot-Welded AA3003-H12 Aluminium Alloy Using a Taguchi Orthogonal Array. *Mater Des* **2014**, *63*, 789–797. https://doi.org/10.1016/j.matdes.2014.07.003.

(10) Arslanoglu, N.; Yigit, A. Investigation of Efficient Parameters on Optimum Insulation Thickness Based on Theoretical-Taguchi Combined Method. *Environ Prog Sustain Energy* **2017**, *36* (6), 1824–1831. https://doi.org/10.1002/ep.12628.

(11) Simpson; James R. Taguchi Techniques for Quality Engineering. *Taylor & Francis*. 1996.

(12) Bademlioglu, A. H.; Canbolat, A. S.; Yamankaradeniz, N.; Kaynakli, O. Investigation of Parameters Affecting Organic Rankine Cycle Efficiency by Using Taguchi and ANOVA Methods. *Appl Therm Eng* **2018**, *145*, 221–228. https://doi.org/10.1016/j.applthermaleng.2018.09.032.

(13) Soni, A.; Patel, R. M.; Kumar, K.; Pareek, K. Optimization for Maximum Extraction of Solder from Waste PCBs through Grey Relational Analysis and Taguchi Technique. *Miner Eng* **2022**, *175*, 107294. https://doi.org/10.1016/j.mineng.2021.107294.

(14) Palanikumar, K. Experimental Investigation and Optimisation in Drilling of GFRP Composites. *Measurement* **2011**, *44* (10), 2138–2148. https://doi.org/10.1016/j.measurement.2011.07.023.

(15) Vidal, C.; Infante, V.; Peças, P.; Vilaça, P. Application of Taguchi Method in the Optimization of Friction Stir Welding Parameters of an Aeronautic Aluminium Alloy. *International Journal of Advanced Materials Manufacturing and Characterization* **2013**, *3* (1), 21–26. https://doi.org/10.11127/ijammc.2013.02.005.
